# Taxonomic and functional characteristics of microbial communities and their correlation with physicochemical properties of four geothermal springs in Odisha, India

**DOI:** 10.3389/fmicb.2015.01166

**Published:** 2015-10-26

**Authors:** Jhasketan Badhai, Tarini S. Ghosh, Subrata K. Das

**Affiliations:** ^1^Department of Biotechnology, Institute of Life SciencesBhubaneswar, India; ^2^TATA Consultancy Services LimitedBhubaneswar, India

**Keywords:** hot springs, metagenomes, microbial diversity, functional characteristics, physicochemical parameters

## Abstract

This study describes microbial diversity in four tropical hot springs representing moderately thermophilic environments (temperature range: 40–58°C; pH: 7.2–7.4) with discrete geochemistry. Metagenome sequence data showed a dominance of *Bacteria* over *Archaea*; the most abundant phyla were *Chloroflexi* and *Proteobacteria*, although other phyla were also present, such as *Acetothermia, Nitrospirae, Acidobacteria, Firmicutes, Deinococcus-Thermus, Bacteroidetes, Thermotogae, Euryarchaeota, Verrucomicrobia, Ignavibacteriae, Cyanobacteria, Actinobacteria, Planctomycetes, Spirochaetes, Armatimonadetes, Crenarchaeota*, and *Aquificae*. The distribution of major genera and their statistical correlation analyses with the physicochemical parameters predicted that the temperature, aqueous concentrations of ions (such as sodium, chloride, sulfate, and bicarbonate), total hardness, dissolved solids and conductivity were the main environmental variables influencing microbial community composition and diversity. Despite the observed high taxonomic diversity, there were only little variations in the overall functional profiles of the microbial communities in the four springs. Genes involved in the metabolism of carbohydrates and carbon fixation were the most abundant functional class of genes present in these hot springs. The distribution of genes involved in carbon fixation predicted the presence of all the six known autotrophic pathways in the metagenomes. A high prevalence of genes involved in membrane transport, signal transduction, stress response, bacterial chemotaxis, and flagellar assembly were observed along with genes involved in the pathways of xenobiotic degradation and metabolism. The analysis of the metagenomic sequences affiliated to the candidate phylum Acetothermia from spring TB-3 provided new insight into the metabolism and physiology of yet-unknown members of this lineage of bacteria.

## Introduction

Terrestrial hot springs represent unique geothermal environments with respect to their geological history, biogeography and physicochemical characteristics that support extremophilic microorganisms (Pace, 1997; Whitaker et al., 2003; Meyer-Dombard et al., 2005). Thermophiles inhabiting high temperature environments are considered to be the closest living relatives of microorganisms present on early Earth (Brock, 1967; Woese et al., 1990; Stetter, 2006). Exploration of microorganisms in geothermal environments has not only provided greater insights into the origin and evolution of earliest life but has also provided access to significant bioresources with potential applications in industries and biotechnology (Huber and Stetter, 1998; Andrade et al., 1999; Satyanarayana et al., 2005; Lewin et al., 2013; López-López et al., 2013).

Culture based studies of the microbial inhabitants of hot springs started with the isolation of thermophilic bacteria by Marsh and Larsen (1953). Over the next several decades, microbial diversity of terrestrial hot springs at different geographical locations across the globe were extensively studied mostly using 16S rRNA based clone libraries in combination with cultivation methods (Ward et al., 1998). Most microorganisms (>99%) are difficult to grow under laboratory conditions, thereby limiting information at the genomic and phenotypic level. In fact, the majority of such organisms remain as unknown (Pace, 1997; Ward et al., 1998; Suenaga, 2012). However, in the last decade, the application of culture-independent genomics or metagenomics (Handelsman, 2004; Sharon and Banfield, 2013) approaches coupled with high-throughput DNA sequencing has proved a promising tool to investigate the population diversity, gene content, function and ecological significance of microbial communities living in diverse hot spring environments (Inskeep et al., 2010, 2013; Swingley et al., 2012; Huang et al., 2013; Satoh et al., 2013; Wang et al., 2013; Delgado-Serrano et al., 2014).

The microbial diversity in hot springs is generally considered to be lower compared to most other environments (Inskeep et al., 2010). However, these microorganisms exhibit remarkable genomic and metabolic flexibility (Segerer et al., 1993; Stetter, 1999; Amend and Shock, 2001; Hamilton et al., 2012; Wemheuer et al., 2013). Significant differences in microbial communities exist among hot springs with different ranges of physicochemical parameters and discrete geographic locations (Meyer-Dombard et al., 2005; Lau et al., 2009; Inskeep et al., 2010, 2013; Swingley et al., 2012; Huang et al., 2013; Mackenzie et al., 2013; Satoh et al., 2013; Wang et al., 2013; Delgado-Serrano et al., 2014). Therefore, analyzing the changes in the diversity and composition of microbial communities in relationship with environmental physicochemical factors across geothermal systems will possibly reveal how microorganisms adapt to and tolerate extreme environmental conditions and increase our understanding of microbial ecology and evolution.

Microbial diversity in the four tropical hot springs located at Atri, Athamallik, Taptapani, and Tarabalo in the state Odisha, India, has received little attention. Cultivation based studies have led to the identification of several new species of bacteria from these hot springs, such as *Comamonas thiooxydans, Gulbenkiania indica, Chelatococcus sambhunathii*, and *Thiomonas bhubaneswarensis* (Panda et al., 2009; Jyoti et al., 2010; Panday and Das, 2010; Narayan et al., 2010). However, comparative taxonomic and functional profiling of the microbial communities in these hot springs has not been performed. Further, these hot springs are situated in two distinct regional geological lineaments and exhibit slightly variable geochemistry (Mahala et al., 2012). These observations prompted us to explore the microbial diversity in these hot springs in detail. We hypothesized that geochemically similar hot springs, regardless of their local climatic conditions at a given time and geographies, have shared microbial communities. In this regard, we employed an integrated approach involving analysis of the environmental physicochemical characteristics and shotgun pyrosequencing of the metagenome. Thus, the present study describes the diversity and composition of four hot spring microbial communities and their relationship with the environmental variables, such as temperature, pH and geochemistry.

## Materials and methods

### Sample collection

**Figure 1:**
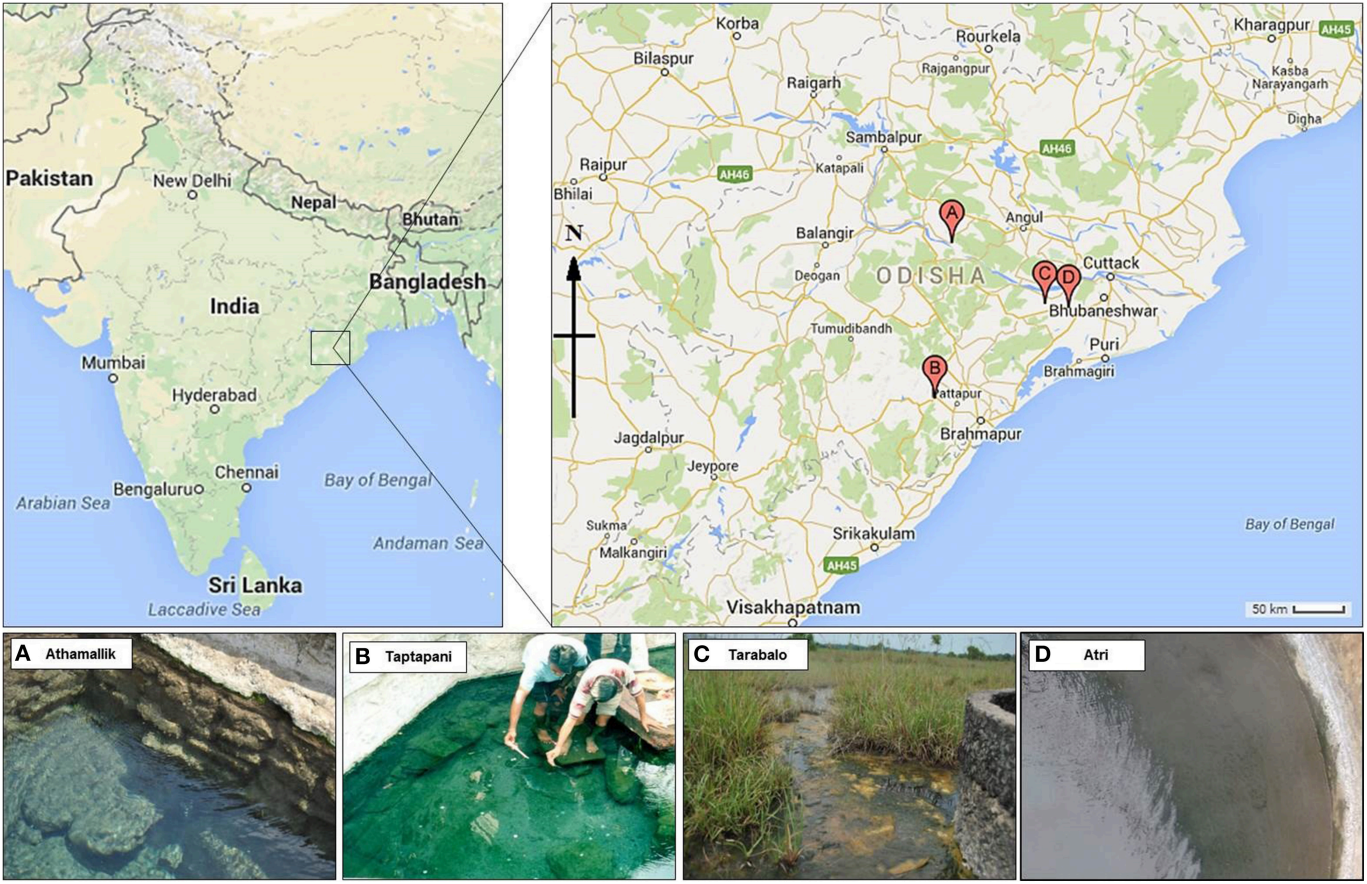
**Geographical location of the four hot springs**. Point A, B, C, and D on the map represent the hot spring Athamallik, Taptapani, Tarabalo, and Atri, respectively.

Samples were collected from four hot springs: Athamallik (HT-1) (20°42′58″N, 84°32′50″E; 80 m altitude) located in the district of Angul, Taptapani (TP-2) (19°29′N, 84°23′45″E; 450 m altitude) in the district of Ganjam, Tarabalo (TB-3) (20°12′20″N, 85°17′50″E; 50 m altitude) in the district of Nayagarh and Atri (AT-4) (20°12′30″N, 85°30′E; 40 m altitude) in the district of Khorda. While HT-1 and TP-2 are geographically widely separated, the TB-3 and AT-4 are situated relatively close to each other (Figure 1). Water temperature (at the main source and in the surrounding areas) was recorded *in situ* using an Enviro-safe thermometer (Sigma, USA) and the pH was measured *in situ* using a portable pH meter (Hanna Instrument, Sigma, USA). For DNA extraction, water and sediment samples were collected in sterile containers from five different spots from the main outlet of each spring where the temperature was almost uniform. After collection samples were pooled by mixing in equal proportions in sterile tubes. For the chemical analysis, springs water (250 ml) were filtered-sterilized (0.22 μm) and stored in −20°C (dry ice) for transport to the laboratory. Chemical properties of the water samples were determined following the guidelines of the Bureau of Indian Standards (IS 3025-1964), Government of India at the Institute of Minerals and Materials Technology, Bhubaneswar, India using standard protocols: total hardness, magnesium, potassium, and calcium were measured using the EDTA titration method; fluoride, and chloride were measured using WTW inoLab pH/ION 735 ion selective electrode; sodium using Systronics flame photometer 128; iron using atomic absorption spectrometry; copper, zinc, arsenic, cadmium, and lead using stripping Voltammetry method on Metrohm 797 VA Computrace; ammonia-nitrogen, nitrate-nitrogen, phosphate-phosphorous, silicate-silicon, and sulfate using Varian Cary 50 Bio UV-visible spectrophotometer; bicarbonate by titration method; dissolved solids using Gravimetry; and conductivity was measured using WTW Multi 340i.

### DNA extraction, sequencing, and data generation

Extraction and purification of total metagenomic DNA from pooled mixes of water and sediment sample was performed using Fast DNA® SPIN Kit for soil (BIO 101, California, USA) following the instructions of the manufacturer with minor modification. Briefly, the silica beads from the Lysing MatrixE of the kit were transferred to a 15 ml sterile Falcon tube, and 2.0 g of wet sediments and 2 ml of lysis buffer (0.12 M sodium phosphate buffer of pH 8.0, 0.5% SDS) were added to it. The tube was vortexed for 3 min, incubated for 65°C for 1 h. After lysis, it was centrifuged at 2300 × g for 20 min and the supernatant was then transferred to a 2.0 ml sterile Eppendorf tube. Following centrifugation at 14,000 × g for 10 min, DNA in the supernatant was purified following the instructions of the manufacturer. DNA was eluted in 50 μl of DES supplied with the kit. Sequencing of the metagenomic DNA samples were done by a commercial source (NxGenBio Life Sciences, New Delhi) applying shotgun pyrosequencing approach on a Roche 454 GS-FLX platform (Roche Applied Sciences, Manheim, Germany) according to the manufacturer's protocol. A total of 11.96, 13.81, 22.79, and 22.6 Mb of sequence data were obtained from the four hot springs: HT-1, TP-2, TB-3, and AT-4, respectively. Raw sequence reads were processed and calculation of DNA sequence statistics such as length and GC content of the processed reads were carried out using the online WebMGA server (http://weizhong-lab.ucsd.edu/metagenomic-analysis/server/) (Wu et al., 2011). The total shotgun metagenomic sequences from each site were preprocessed using the following parameters: [a] quality filtration (min length: 65 bases; and min average quality score: 21), and [b] CD-HIT-454 clustering at a sequence identity threshold of 0.98 to remove artificial duplicates generated during 454 sequencing (Niu et al., 2010). Subsequently, after the preprocessing, 25307, 29164, 48251, and 47409 high quality reads were retained for Athamallik (HT-1), Taptapani (TP-2), Tarabalo (TB-3), and Atri (AT-4) metagenome, respectively for further analyses (Supplementary Table 1).

### Taxonomic and functional analysis of metagenomic sequences

Taxonomic and functional assignments for the protein-coding gene sequences in each metagenomic reads data set were obtained using BLASTX (Altschul et al., 1997) (applying an *e*-value cutoff of 1e–10) against NCBI-NR database (local BLAST−2.2.29+ package, built: July 30, 2014) (BLAST® Help, 2008; Camacho et al., 2009) and the standalone MEtaGenome analyzer software (MEGAN v5.5.3) (Huson et al., 2011) according to suggested parameters for the lowest common ancestor (LCA) assignment algorithm (min score: 50.0; max expected: 0.01; top percent: 10.0; and min support percent: 0.01). To perform taxonomic analysis, the MEGAN5 program placed the BLASTX annotated genes/reads onto the NCBI taxonomy tree (Jun 24, 2014). To perform functional analysis, MEGAN5 mapped the BLASTX annotated genes/reads having matches to RefSeq ids onto the SEED (Overbeek et al., 2005) and KEGG (Kanehisa and Goto, 2000) classification tree using SEED (May 17, 2010) and KEGG (Dec 1, 2010) identifiers, respectively. Additionally, taxonomic and functional annotation was carried out on the WebCARMA v1.0 online system (http://webcarma.cebitec.uni-bielefeld.de/cgi-bin/webcarma.cgi) (Gerlach et al., 2009).

### Statistical analysis of data

For all statistical analyses, gene counts were normalized by dividing the number of gene hits to individual taxa/functional role by total number of gene hits in each metagenome dataset to remove bias due to difference in sequencing efforts. To identify microbial taxa/function significantly over-represented or under-represented in each metagenome, single sample vs. all other samples statistical tests of the relative gene abundances (obtained from MEGAN5 classification profiles) were carried out applying two-sided Welch's *t*-test with Benjamini-Hochberg *False Discovery Rate (FDR)* multiple test correction method and a *P* < 0.05 using the STAMP v2.0.8 software (Parks and Beiko, 2010; Parks et al., 2014). Multivariate principal component analysis (PCA) of 22 physicochemical parameters i.e., temperature, pH, conductivity, total hardness (CaCO_3_), calcium, magnesium, sodium, potassium, chloride, ammonia-nitrogen, nitrate-nitrogen, fluoride, phosphate-phosphorous, bicarbonate (HCO${}_{3}^{-}$), sulfate, silicate-silicon, iron, lead, cadmium, zinc, arsenic, and dissolved solids were carried out to determine which environmental variables best explained the observed community patterns. Before the multivariate analyses, all variables were normalized by subtracting the mean of the raw data and dividing by the standard deviation of the raw data to better conform to normality (Pagaling et al., 2009). Stepwise canonical correspondence analysis (CCA) was carried out to analyze the relationships between the 22 environmental factors and normalized abundances of major taxonomic/functional groups respectively. Rarefaction curves based on KEGG Orthology Identifiers (KO-Id) were used to estimate functional richness of the metagenomic data sets. The rarefaction curves, multivariate PCA and CCA plots were generated using the PAST v3.02 software (Hammer et al., 2001). The influence of the environmental variables on the relative abundances of major genera was also investigated using the Partial Least Square (PLS) regression technique (Esposito Vinzi et al., 2010). The PLS regression analysis was performed separately for each individual genus, taking the 22 environmental variables as predictors. The *P*-value of this correlation indicates the statistical significance of this association. Furthermore, to take into account the false detection rate, we have also computed the power of the correlation tests with *P* < 0.05. Those genera, for which the PLS components were observed to have correlations with *P* < 0.05 and *Power* > 0.5 with their abundances, were identified to be significantly influenced by the environmental variables. Two-way hierarchical clustering analyses were used to compare the row-standardized (by subtracting the mean of each functional group across samples) relative gene abundance data using the Pearson correlation (uncentered) metrics and average-linkage clustering method on CLUSTER v3.0 software (http://bonsai.hgc.jp/~mdehoon/software/cluster/software.htm) (de Hoon et al., 2004). The dendrogram were visualized using Java Tree View v1.1.6r4 software (http://jtreeview.sourceforge.net/).

### Sequence data availability

All four metagenomic sequence data sets and corresponding metadata are available on the Metagenomic RAST (MG-RAST) server (Meyer et al., 2008) under project IDs: 4555635.3, 4555636.3, 4555637.3, and 4555638.3.

## Results and discussions

### Physicochemical characteristics of the hot springs

The temperature and pH of the four hot springs were recorded in the range of 42–58°C and 7.2–7.4. The highest temperature was recorded at Atri (58°C) followed by Tarabalo (57°C) and Athamallik (56°C), whereas the lowest temperature was recorded at Taptapani (42°C). The aqueous concentrations of major ions such as sodium, potassium, calcium, magnesium, fluoride, chloride, ferric iron, arsenic, nitrate, silicate, carbonate/bicarbonate, phosphate, and sulfate were varied among the springs (Table 1). A principal component analysis (PCA) of the physicochemical parameters showed the four hot springs were separated into three geochemically distinct habitats (Supplementary Figure 1). The first two principal components explained 87.65% of the total variance. The first component separated the low temperature in spring TP-2 from the other three high temperature springs. The second component separated the less oligotrophic spring HT-1 from the other three relatively more oligotrophic springs (as indicated by the low levels of dissolve nitrogen, phosphorus, and total dissolve solids). Overall, HT-1 had the highest aqueous concentration of CaCO_3_ (total hardness), calcium, magnesium, and sulfate, while TP-2 had distinctly high concentration of fluoride, chloride, sodium, potassium, arsenic, iron, and lead. The water samples from other two high temperature springs (TB-3 and AT-4) were relatively similar with small variation in the concentrations of sodium, fluoride, chloride, dissolved solids, and conductivity (Supplementary Figure 1). These observed differences and/or similarities in the physicochemical parameters were predicted to be due to the differences in geological substrate as well as the geographies and separation between the four hot springs.

**Table 1:** **Physicochemical parameters of the four hot springs**.

**Sample ID**	**HT-1**	**TP-2**	**TB-3**	**AT-4**
Hot spring	Athamallik	Taptapani	Tarabalo	Atri
Total hardness (CaCO_3_), mg/L	145.5	25	18	48
Calcium, mg/L	54.5	10	6	18
Magnesium, mg/L	3.7	ND	1.2	1.2
Sodium, mg/L	109	195.5	90.1	36.9
Potassium, mg/L	4.7	13.5	2.8	1.4
Chloride, mg/L	173	249.8	143.5	10.6
Ammonia-Nitrogen, mg/L	3.9	4.3	3.6	3.4
Nitrate-Nitrogen, mg/L	5.2	3.61	6.5	4.7
Fluoride, mg/L	3.8	9.1	11.48	4.04
Phosphate-Phosphorous, mg/L	3.9	3.7	3.1	2.3
Bicarbonate (HCO${}_{3}^{-}$), mg/L	185	78.1	201	161
Sulfate, mg/L	85.2	71	44	29.4
Silicate-Silicon, mg/L	4.9	4.2	2.3	3.5
Iron, mg/L	0.17	0.23	0.21	0.11
Copper, mg/L	<0.01	<0.01	<0.01	<0.01
Lead, ppb	3.7 x 10^−4^	31.60 x 10^−3^	15.76 x 10^−3^	8.2 x 10^−4^
Cadmium, mg/L	2.3 x 10^−4^	22.73 x 10^−3^	11.53 x 10^−3^	ND
Zinc, ppb	1.36 x 10^−3^	2.3 x 10^−3^	4.5 x 10^−3^	3.12 x 10^−3^
Arsenic, ppb	1.23 x 10^−3^	2.32 x 10^−3^	1.234 x 10^−3^	6.99 x 10^−4^
Conductivity, μs/cm	1131	1033	744	380
Dissolved Solids, mg/L	780.4	647	510	271
Temperature range (°C)	56	42	57	58
pH	7.4	7.2	7.3	7.4

*ND, not detected*.

### Microbial community compositions and diversity

Comparative analysis of the four hot spring microbial communities revealed that approximately 56.2% of the total sequence reads in each metagenomic data sets were taxonomically classified (Supplementary Table 1). The majority of the sequence reads were classified as *Bacteria* (54.5%) followed by *Archaea* (1.7%) and *Eukarya* (< 0.1%). In addition, there were sequence reads that could not be assigned to known taxa (43.8%) due to the lack of reference sequences and/or use of a strict LCA-taxonomic assignment algorithm. This MEGAN5 taxonomic distribution was consistent with the result obtained from WebCARMA online server based taxonomic annotation pipeline (data not presented). This analysis also suggested that these hot springs are a potential reservoir of diverse novel microorganisms which are “yet unknown” or uncharacterized. A total of 30, 30, 32, and 36 individual phyla were identified in the samples HT-1, TP-2, TB-3, and AT-4, respectively. Within the domain *Bacteria*, only 66.5% of the assigned sequences were classified at the phylum level, and there was high abundance of the phyla *Chloroflexi* and *Proteobacteria*, although other major phyla were also abundant (in decreasing order of abundance), e.g., Acetothermia (specifically abundant in TB-3, >37%), *Nitrospirae, Acidobacteria, Firmicutes, Deinococcus-Thermus, Bacteroidetes, Thermotogae* (not detected in TP-2), *Verrucomicrobia, Ignavibacteriae, Cyanobacteria* (specifically abundant in TP-2, >15%), *Actinobacteria, Planctomycetes, Spirochaetes, Armatimonadetes*, and *Aquificae*. Besides, several minor bacterial groups (abundance < 1% of all assigned bacterial sequences), such as *Chlorobi, Deferribacteres, Dictyoglomi, Marinimicrobia, Gemmatimonadetes, Synergistetes, Thermodesulfobacteria*, including some newly proposed phyla and/or candidate divisions (Rinke et al., 2013; Hedlund et al., 2014), such as Aminicenantes, Atribacteria, Calescamantes, “*Candidatus* Saccharibacteria,” Cloacimonetes, Fervidibacteria, Hydrogenedentes, Latescibacteria, and Poribacteria were also observed in some samples. Likewise, within the domain *Archaea* about 79% of the assigned sequences were classified at the phylum level. In all samples, the phylum *Euryarchaeota* was found to be most abundant followed by phylum *Crenarchaeota* (not detected in TP-2), whereas sequences affiliated to *Thaumarchaeota* and *Korarchaeota* were observed at low abundances in two samples (TB-3 and AT-4).

**Figure 2:**
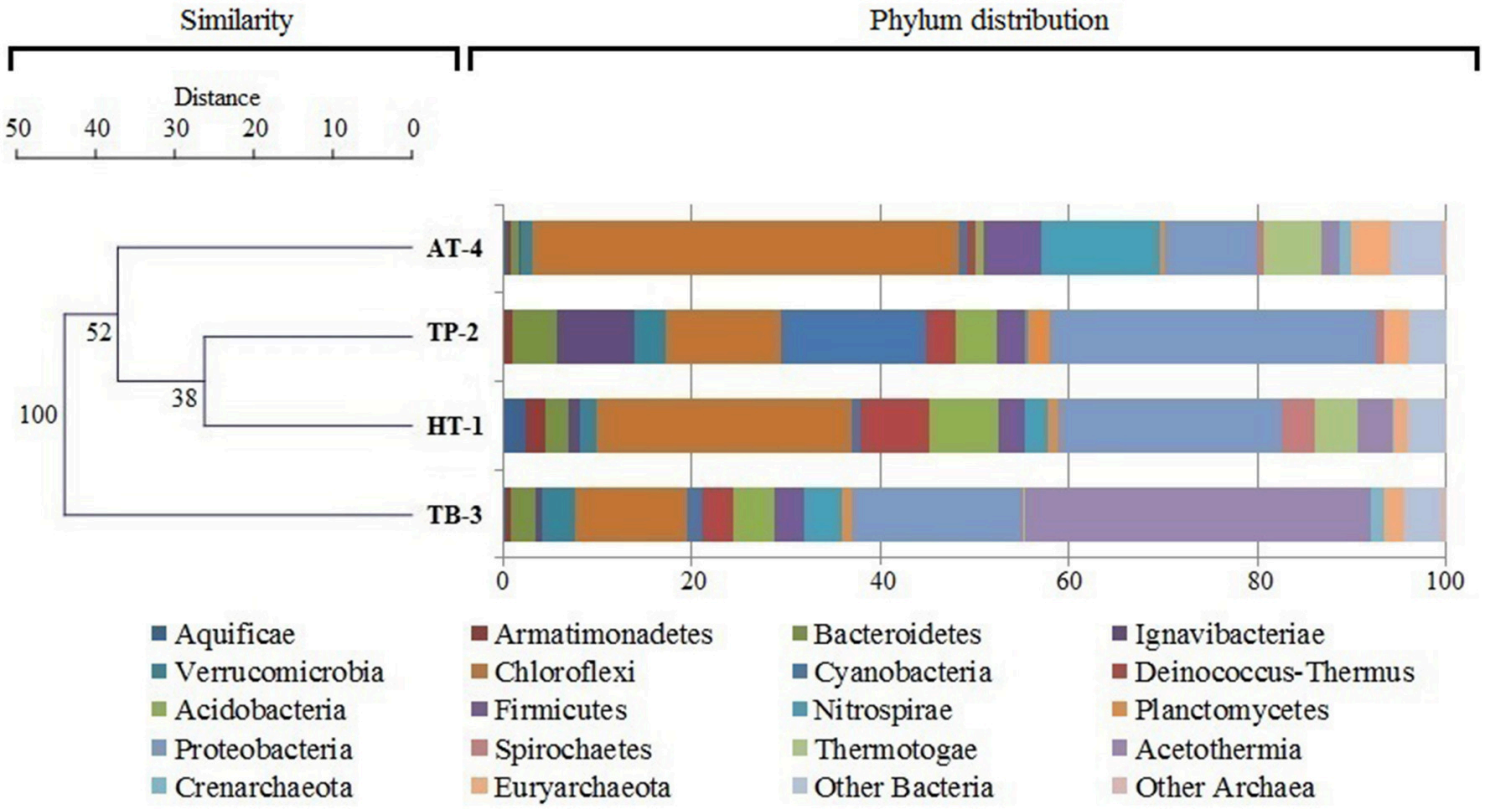
**Relative distribution of microbial phyla (both bacterial and archaeal) and hierarchical clustering of metagenomes**. The phylum abundance data was obtained from MEGAN5 taxonomic classification of the protein-coding sequences in the metagenomes: HT-1 (Athamallik), TP-2 (Taptapani), TB-3 (Tarabalo), and AT-4 (Atri). The hierarchical clustering of the metagenomes was based on unweighted pair-group average (UPGMA) algorithm and Euclidean similarity index with 1000 bootstrap replicates.

**Figure 3:**
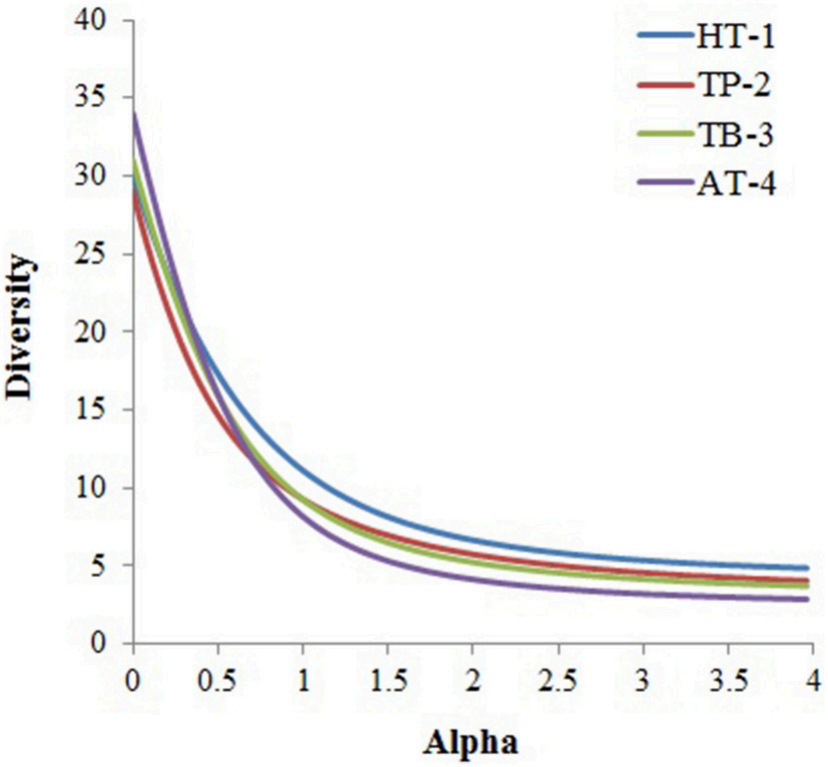
**Alpha diversity was calculated for each community based on taxa distribution at phylum level using the PAST statistical program**. The phylum abundance for each metagenome was obtained from MEGAN5 taxonomic classification of the metagenomes.

Although, the four microbial communities shared several taxonomic groups at the phylum level, they differed significantly with respect to the overall phyla composition (Figure 2, Supplementary Table 2) and diversity (Figure 3). These similarities/dissimilarities were likely due to the differences in the geographical features and the discrete environmental physicochemical conditions of the springs. Differences in the microbial communities were even more evident at lower taxonomic ranks (Supplementary Figure 2), and the most diverse lineages observed were *Chloroflexi, Proteobacteria, Acidobacteria, Firmicutes, Bacteroidetes*, and *Euryarchaeota*. Although, the phyla *Proteobacteria, Acidobacteria* and *Bacteroidetes* are ordinarily not among the dominant and diverse lineages in hot springs (Bolhuis et al., 2014), and are ancestrally marine lineages (Battistuzzi and Hedges, 2009), they dominated the hot springs surveyed in this study (except AT-4). These bacteria have been detected in sediments/soils in terrestrial environments (Quaiser et al., 2003; Roesch et al., 2007). Thus, the observed high abundance of these bacteria in this study might be due to the sourcing from nearby sediment/soil communities surrounding the hot springs.

The abundant distribution of sequences taxonomically affiliated to the members of phototrophic bacteria from the phyla *Cyanobacteria* (orders *Chroococcales*, and *Oscillatoriales*), *Chloroflexi* (order *Chloroflexales), Acidobacteria* (genus “*Candidatus* Chloracidobacterium”), *Chlorobi* (order *Chlorobiales*), and *Gemmatimonadetes* (genus *Gemmatimonas*), *Alphaproteobacteria* (families *Bradyrhizobiaceae, Rhodobacteraceae*, and *Rhodospirillaceae*), *Betaproteobacteria* (genus *Rubrivivax* and *unclassified Rhodocyclaceae*), and *Gammaproteobacteria* (orders *Chromatiales*) (Bryant and Frigaard, 2006; Bryant et al., 2011; Zeng et al., 2014) predicted that photoautotrophic carbon fixation might be one of the most important biosynthetic process in these oligotrophic habitats.

A comparison of the microbial communities at phylum level in these Indian hot springs and those in other tropical hot springs located in Philippines, China, and Malaysia revealed that the composition of the dominant groups were largely distinct from the others, with few similarities also observed. The microbial communities in the hot springs in Philippines (Huang et al., 2013) were dominated by *Aquificae* and *Firmicutes*, except the BAG-2 hot spring which showed a composition of major groups similar to that in our study and was dominated by the phyla *Proteobacteria, Chloroflexi, Acidobacteria, Thermotogae, Verrucomicrobia*, and *Firmicutes*. Whereas, the hot springs in Yunnan in China (Song et al., 2013) were dominated by *Proteobacteria, Aquificae, Thermotogae, Bacteroidetes, Firmicutes, Deinococcus-Thermus*, and *Thermodesulfobacteria*. The moderate temperature hot springs in Tibetan Plateau in Northwest China (Wang et al., 2013) were dominated by *Aquificae, Crenarchaeota, Chloroflexi*, and *Deinococcus-Thermus*. The hot spring in Sungai Klah in Malaysia (Chan et al., 2015) was dominated by the *Firmicutes, Proteobacteria, Aquificae, Verrucomicrobia, Thermotogae, Ignavibacteriae*, and *Euryarchaeota*. While the four hot springs in India were characterized by moderate temperature (42–58°C), near-neutral pH, and moderate salinity, the hot springs in Philippines (except BAG-2 which had conditions similar to that in the Indian springs), China, and Malaysia had high temperature (50–110°C), acidic or alkaline pH, and low to high salinities. Therefore, the observed dissimilarities/similarities of the dominant taxonomic groups or phyla across the compared tropical hot springs were likely due to the differences in the physicochemical parameters, such as temperature, pH, and salinity.

### Microbial community composition corresponding to the environmental conditions

In general, microbial populations may differ due to inherent differences in the physicochemical parameters and the geographical surroundings of the hot springs. The temperature in TP-2 was relatively lower as compared to the other three hot springs. This might have facilitated the survival of relatively mesophilic photosynthetic and/or photoautotrophic bacteria belonging to the phyla *Cyanobacteria* (*Chroococcales, Oscillatoriales, Nostocales, Pleurocapsales*, and *Stigonematales*) as found in other aquatic environments with neutral or slightly alkaline pH (Miller et al., 2009; Klatt et al., 2011; Wang et al., 2013). Similarly, predominance of the recently proposed candidate phylum Acetothermia (Hugenholtz et al., 1998; Takami et al., 2012) in TB-3 sample could be due to specific geochemistry of the spring. Our analysis revealed taxonomic richness of the microbial community in the samples collected from the low temperature spring (TP2) was more (61 distinct taxa), whereas in the other three high temperature springs was relatively less (47–53 distinct taxa) (Supplementary Figure 3). This observation reiterates that with the increase in temperature of the habitat, the taxonomic richness and diversity of the resident microbial community decreases (Meyer-Dombard et al., 2005; Miller et al., 2009; Inskeep et al., 2010; Tobler and Benning, 2011; Swingley et al., 2012; Cole et al., 2013; Wang et al., 2013; Sharp et al., 2014). Moreover, within the same phyla, organisms belonging to specific lineages that occupy sites with discrete environmental gradients may demonstrate divergent phenotypic characteristics (Omelchenko et al., 2005). The current study identified four major genera: *Chloroflexus, Roseiflexus, Anaerolinea*, and *Caldilinea* in all springs. Despite of belonging to the same taxonomic group (phylum *Chloroflexi*), the abundances and distributions of these anoxygenic photoheterotrophs (*Chloroflexus* and *Roseiflexus*) and chemoorganotrophs (*Anaerolinea* and *Caldilinea*) followed discrete patterns. While *Chloroflexus* and *Anaerolinea* were dominant in the HT-1, *Roseiflexus* and *Chloroflexus* were dominant in the TB-3 and AT-4, respectively, and *Anaerolinea* was dominant in the TP-2. Differential habitat preference was also observed for the genera *Meiothermus* and *Thermus* within the class *Deinococci*. While *Meiothermus* was highly represented in TP-2 and TB-3, the *Thermus* was specifically highly represented in HT-1. The likely reasons for these habitat preferences could be the functional differences in carbon metabolism or differences in electron donor utilization. Moreover, the specific abundance of these genera is likely to be influenced by the geochemical characteristics of the corresponding hot springs.

**Figure 4:**
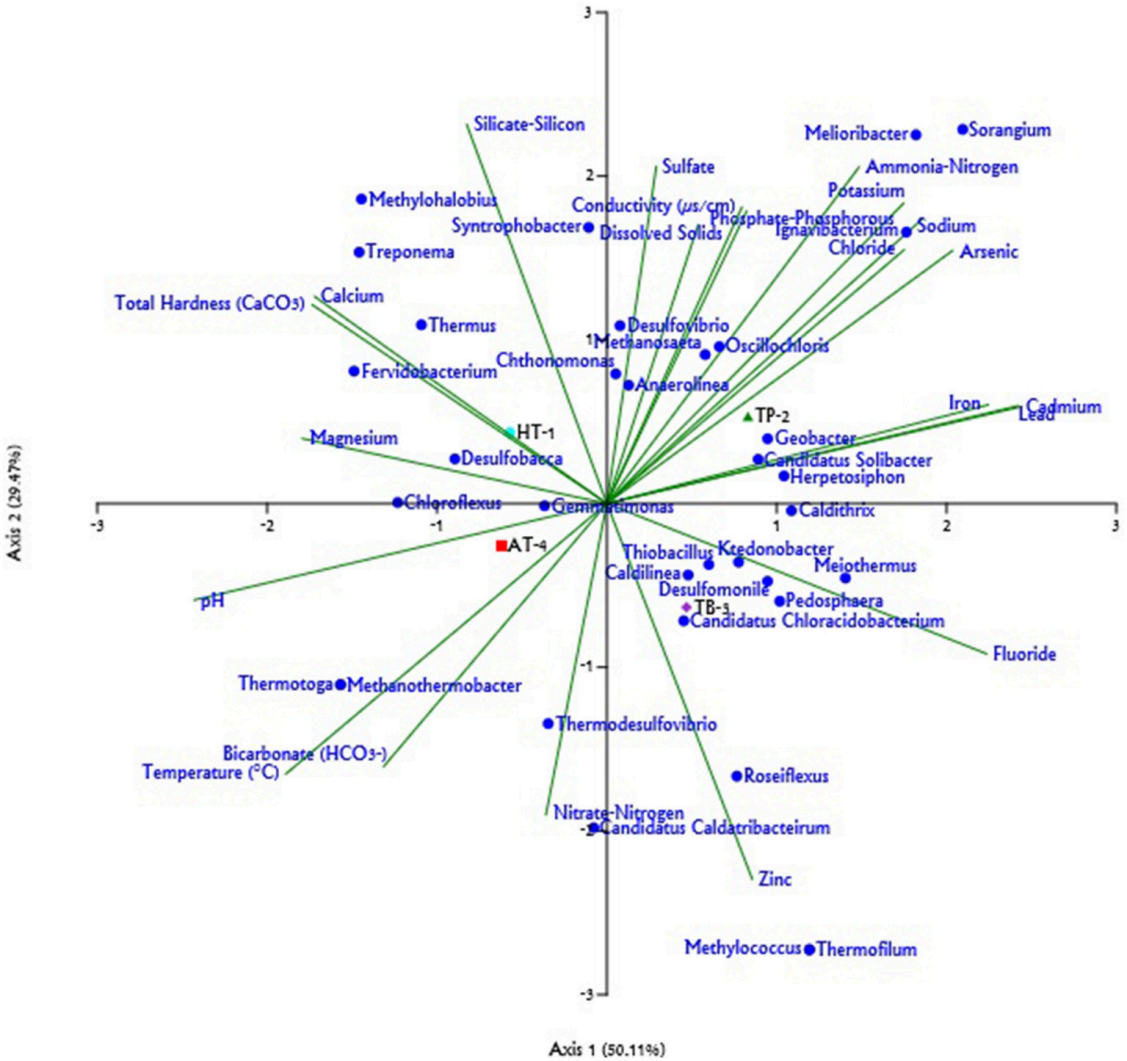
**Triplot generated for the Canonical Correspondence Analysis (CCA) of relative abundance of different genera (only those with >1% represented in at least one sample) and 22 physicochemical parameters across hot springs**. The physicochemical parameters are represented by green lines; the genera are shown as blue circles; and the hot springs: Athamallik (HT-1), Taptapani (TP-2), Tarabalo (TB-3), and Atri (AT-4) are shown as colored symbols. The length of line indicates which physicochemical parameters most strongly determined the genera distribution.

Analysis of the major genera represented across metagenome samples and evaluation of their association with the environmental physicochemical parameters of the hot springs based on CCA predicted that the abiotic environment of the springs had a strong influence over the microbial community composition. Further, a partial least square (PLS) based regression analysis revealed that the some of the physicochemical parameters of the environments were significantly correlated (*P* < 0.05, *Power* > 0.5) with the abundances of 13 out of the 22 major genera observed to be present across at least three hot springs (Supplementary Table 3). Analysis of the metagenome collected from Athamallik (HT-1) identified a total of 20 genera which were over- and/or under-represented (Supplementary Figure 4A). The CCA and PLS analyses showed that the abundance of the genera *Thermus, Syntrophobacter, Anaerolinea, Chthonomonas*, and *Chloroflexus* was positively correlated with the aqueous concentration of magnesium, silicate-silicon, and total hardness (Figure 4, Supplementary Figure 5). Similarly, analysis of the metagenome from Tarabalo (TB-3) showed an over-representation of the genera *Roseiflexus, Meiothermus, Pedosphaera, Methylococcus*, and “*Candidatus* Chloracidobacterium” (Supplementary Figure 4B). Extending the CCA and PLS analyses to this sample revealed that the abundance of genera *Roseiflexus* and “*Candidatus* Chloracidobacterium” was strongly correlated with the aqueous concentration of fluoride and zinc, whereas the genera *Meiothermus* and *Pedosphaera* were positively correlated with the fluoride, chloride, and metal ions, such as sodium, potassium, iron, lead, and cadmium (Figure 4, Supplementary Figure 5). Whereas, in the sample from Atri (AT-4), having the highest temperature (58°C) among the four hot springs, the genera *Chloroflexus, Thermodesulfovibrio, Thermotoga, Desulfobacca*, and *Methanothermobacter* were observed significantly over-represented (Supplementary Figure 4C). Notably, the CCA and PLS analyses revealed that the abundances of these genera were also positively correlated with the temperature of the hot springs. In the same analysis, it was observed that the abundance of genus *Thermodesulfovobrio* positively correlated with the aqueous concentrations of nitrate-nitrogen and bicarbonate (Figure 4, Supplementary Figure 5). Similarly, in the Taptapani (TP-2) hot spring, it was observed that the population of the genera *Ignavibacterium* and *Melioribacter, Methanosaeta, Sorangium*, and *Geobacter* (Supplementary Figure 4D) were significantly high. The CCA and PLS analysis also revealed that the abundances of these genera were positively associated with aqueous concentrations of sodium, potassium, fluoride, chloride, (properties which were observed to be the highest for TP-2; Figure 4, Supplementary Figure 5).

### Functional analysis of the metagenome

Functional analysis of the metagenome showed approximately 27% and 36.2% of the predicted protein-coding sequences or genes were matched to SEED subsystems and KEGG pathways, respectively (Supplementary Table 1). At the level of SEED classification, in all samples, approximately 24.4% of the genes were related the subsystems of carbohydrate and protein metabolism whereas, 14.6% of the sequences were related to subsystems of nucleic acid metabolism, and nucleosides and nucleotides metabolism. About 8.4% and 6.2% of the genes were related to subsystems of amino acids and derivatives, and cofactors, vitamins, prosthetic groups, and pigments respectively. While 7.2% of the genes were related to subsystems of respiration, only < 0.1% genes were related to subsystems of photosynthesis. A relatively high number of the genes were related to the subsystems of virulence (8.0%) in all the metagenomes, whereas 5.5, 3.0 and 2.2% of the predicted protein sequences were related to the subsystems of cell wall and capsule, stress response, motility, and chemotaxis, respectively. An additional 1.7% of the genes were related to subsystems of regulation and cell signaling (Supplementary Table 4).

The KEGG mapping and subsequent classification of the predicted protein sequences identified 2304, 2435, 2659, and 2242 unique genes (KEGG Orthology identifiers, KO-Ids) from metagenome of HT-1, TP-2, TB-3, and AT-4 respectively (Supplementary Table 5). The majority of the predicted protein sequences were associated with the functions related to metabolism (49.2%) followed by genetic information processing (9.4%) and environmental information processing (6.8%). Also, a large proportion (>30%) of the identified KEGG Orthologous genes remained uncategorized. A rarefaction analysis based on KO-Ids revealed that the low temperature spring TP-2 was functionally rich compared to the other three high temperature springs (Supplementary Figure 6), which indicates the influence of temperature in regulating microbial community composition and function in geothermal habitats.

The majority of the sequence hits to functional genes were phylogenetically affiliated to the members of the numerical dominant moderately thermophilic bacteria of the phyla *Chloroflexi, Proteobacteria, Acidobacteria, Bacteroidetes*, and *Firmicutes*, as well as *Archaea* of the phyla *Euryarchaeota* and *Crenarchaeota*; correspondingly, carbon and sulfur cycling were predicted to be the key physiological functions in these oligotrophic hot spring environments. While members of *Chloroflexi* (genera *Chloroflexus* and *Roseiflexus*) are predicted to be primarily involved in photoautotrophic carbon fixation (Bryant et al., 2011; Klatt et al., 2013), the members of *Proteobacteria* (especially Gamma-, and Delta- lineages) are predicted to be mainly involved in the sulfur cycling (Bryant and Frigaard, 2006; Yamamoto and Takai, 2011; Bolhuis et al., 2014). Moreover, the sulfate reducing *Proteobacteria* are important decomposers of organic matter under anoxic conditions in the hot spring environments. The abundantly detected bacteria of the genus *Thermodesulfovibrio* (phylum *Nitrospirae*) are also important sulfate-reducers in these thermal springs. The heterotrophic *Bacteroidetes* and *Firmicutes* (orders *Bacillales, Clostridiales*, and *Thermoanaerobacterales*) are predicted to be important decomposers of organic matter and involved in carbon cycling (Harris et al., 2013; Bolhuis et al., 2014). Further, the photoheterotrophic *Acidobacteria* (genus “*Candidatus* Chloracidobacterium”) and the chemoorganotrophic *Chloroflexi* (genera *Anaerolinea* and *Caldilinea*) are also predicted to be important players in carbon cycling.

Although, genes predicted to be involved in carbon, nitrogen, and sulfur metabolism pathways were identified (based on both SEED and KEGG classification) in this study, no specific marker genes related to methanogenesis, and sulfur oxidation or reduction has been identified. However, gene sequences belonging to novel microorganisms were abundant in these hot springs (approximately 44% of the metagenomic sequences had no significant match with sequences from any known microorganisms), and their functions remain to be elucidated.

The annotated carbon metabolism genes included those involved in pathways of carbohydrate degradation, carbon fixation and methane metabolism. Analysis of the carbohydrate degradation pathways identified genes predicted to be encoding enzymes, such as: alpha-amylase, glucoamylase, and beta-xylosidase (starch degradation); endoglucanase, alpha-glucosidase, and beta-glucosidase (cellulose degradation); alpha-arabinofuranosidase (hemicellulose degradation); chitinase, hexosaminidase, alpha-mannosidase, beta-mannosidase and alpha-fucosidase (chitin and glycan degradation). The carbon fixation pathways in these springs were predicted to be driven by all the six known autotrophic pathways (Supplementary Table 6): (a) the reductive pentose phosphate pathway in *Cyanobacteria* (orders *Chroococcales* and *Oscillatoriales*) and *Alphaproteobacteria* (family *Rhodospirillaceae*); (b) the reverse tricarboxylic acid cycle in *Aquificae* (genus *Hydrogenobacter*), *Chlorobi* (order *Chlorobiales*), *Deltaproetobacteria, Nitrospirae* (genus *Thermodesulfovibrio*), and Euryarchaeota (*Methanosaeta* and *Methanothermobacter*); (c) the reductive acetyl-CoA pathway in *Acetothermia* (Takami et al., 2012), *Deltaproetobacteria* (family *Chromatiaceae*), *Acidobacteria* (genus “*Candidatus* Chloracidobacterium”), *Deinococci* (genera *Meiothermus* and *Thermus*), *Firmicutes, Planctomycetes, Spirochaetes*, and methanogenic *Euryarchaeota* (family *Methanosaetaceae*); (d) the 3-hydroxypropionate bicycle in *Chloroflexaceae* (Zarzycki et al., 2009); (e) the 3-hydroxypropionate/4-hydroxybutyrate cycle in *Crenarchaeota* (order *Sulfolobales*) in the spring AT-4, the identified marker genes for which were methylmalonyl-CoA mutase, and the bifunctional propionyl-CoA carboxylase (Hügler et al., 2003; Berg et al., 2007); and (f) the dicarboxylate-hydorxybutyrate cycle in *Crenarchaeota* (affiliated to the orders *Desulfurococcales* and *Thermoproteales*) in the springs TB-3 and AT-4 (Huber et al., 2008).

Furthermore, analysis of the metagenome sequences affiliated to methane metabolism pathways predicted genes involved in methanogenesis utilizing carbon dioxide, acetate, and methylamine. Although, the identified methane metabolism genes were not specific to the pathway of methanogenesis, there taxonomic affiliation to the thermophilic methanogens of the archaeal classes *Methanobacteria* (order *Methanobacteriales*) and *Methanomicrobia* (orders *Methanomicrobiales* and *Methanosarcinales*) suggested that methanogenesis could be occurring in these hot springs.

Analysis of the metagenome sequences involved in nitrogen metabolism identified genes involved in the pathways for the assimilatory nitrate reduction (*nasA* and *nirA*), dissimilatory nitrate reduction (*narGHI, nirB*, and *nrfA*), denitrification (*nirK, norB*, and *nosZ*), nitrogen fixation (*nifDKH*) and nitrification (*hao*). These results were consistent with the taxonomic abundance of the nitrogen metabolizing bacteria, such as members of the phyla *Proteobacteria* (Alpha-, Beta, and Gamma- lineages), and *Nitrospirae* (genus *Thermodesulfovibrio*) across the four springs.

In microorganisms many different mechanisms exist to deal with low nutrients availability in the environment, such as using ABC transporters and two-component systems. Consistent with the low phosphate-phosphorous availability across the four hot springs, gene encoding proteins predicted to be involved in phosphate-recycling mechanisms, such as the high-affinity phosphate transporters (*pstA, pstB, pstC*, and *pstS*) and phosphonate transporters (*phnC, phnD*, and *phnE*), and the phosphate limitation sensing two-component systems (*phoR, phoA, phoP, phoA, phoD*, and *regX3*) were detected across all samples. Also, gene sequences encoding for the transporters of branched-chain amino acids transport system, peptide/nickel transporters (*oppABCDF* operon), cobalt/nickel transport system (*cbiM, cbiQ*, and *cbiO*), iron complex transport system (*fhuD, fhuB*, and *fbuC*), and zinc transporters (*znuA, znuB*, and *znuC*) were detected across all metagenomes. Furthermore, genes encoding pathways to deal with low nitrogen availability in the springs were also detected including the ABC transporters genes involved in nitrate transports (*nitT* family of transporters gene) and the two-component system (*glnA, glnB, glnD, glnG, ntrXY*, and *nifA*).

A deeper analysis of the SEED subsystems related to respiration revealed several putative genes involved to anaerobic respiration in all the metagenomes which was consistent with the availability of various terminal electron acceptors, such as ferric iron, nitrate, sulfate, and arsenate, etc. in these hot spring environments. Similarly, the observed high occurrences of genes related to cell wall and capsule, stress response, motility, and chemotaxis, and regulation and cell signaling (Supplementary Table 4) compared to the microbial metagenomes from other niches (Dinsdale et al., 2008), suggested that these functions might be important in adaptive responses of the microorganisms to changes in environmental conditions and low nutrient availabilities in hot springs.

**Figure 5:**
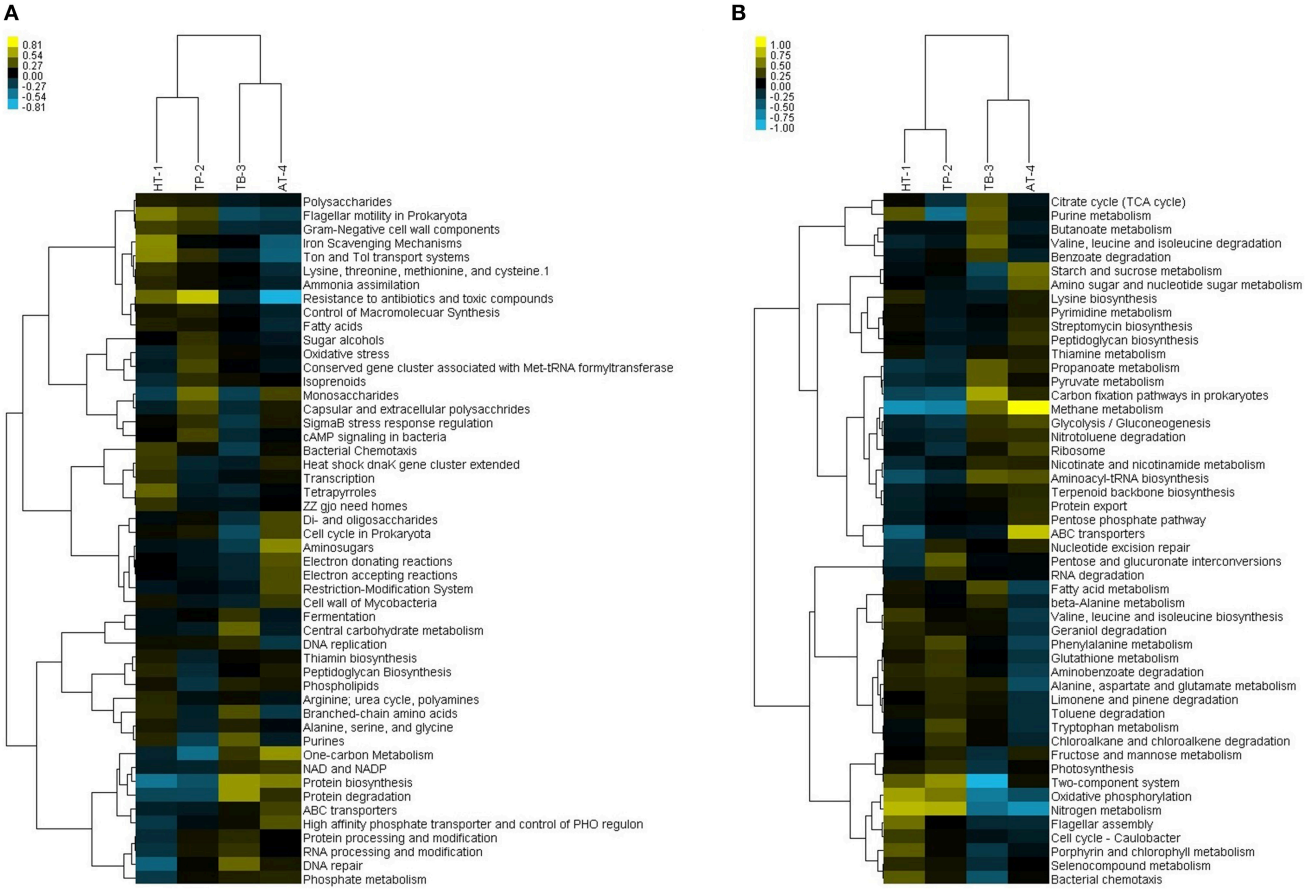
**Two-way hierarchical clustering analyses of the hot springs vs. metagenomes based on relative percentage of gene sequences with matches to different SEED subsystems and KEGG pathways. (A)** Clustering of hot springs vs. SEED subsystem functions. Subsystems with a standard deviation greater than 0.1 of the observed values, having at least three samples with ≥0.4% of the total sequences are shown. **(B)** Clustering of hot springs vs. KEGG pathways. Pathways with a standard deviation greater than 0.1 of the observed values, having at least three samples with ≥0.3% of the total sequences are shown.

The differences and/or similarities among the four springs were evaluated using two-way hierarchical clustering analyses. However, only small variations were observed in the relative abundances and distribution of various functional subsystems/pathways. The clustering analysis of the SEED subsystems showed two distinct groups, with HT-1 and TP-2 in one group and TB-3 and AT-4 in the other group. This clustering was consistent with the separation observed in PCA of the relative abundances of SEED categories (Supplementary Figure 7). The top 50 SEED subsystems that significantly contributed to the variability across the four metagenome samples were further analyzed. The more highly represented SEED subsystems in the first cluster (HT-1/TP-2) corresponded to resistance to antibiotics and toxic compounds, flagellar motility in Prokaryota, Ton and Tol transport systems, iron scavenging mechanisms, fatty acids, Gram-negative cell wall components, and control of macromolecular synthesis, whereas those highly represented in the second cluster (TB-3/AT-4) corresponded to protein biosynthesis, protein degradation, one-carbon metabolism, DNA repair, ABC transporters, NAD and NADP, high affinity phosphate transporter, control of PHO regulon and phosphate metabolism (Figure 5A).

The hierarchical clustering analysis of KEGG pathways also revealed a similar result to that observed at SEED subsystems level where the clustering occurred between HT-1 and TP-2 in one hand and TB-3 and AT-4 in the other (Figure 5B). While HT-1/TP-2 cluster showed high occurrences of pathways linked to photosynthesis, nitrogen metabolism, aromatic amino acids metabolism and xenobiotics degradation and metabolism, cell motility, and signal transduction, the TB-3/AT-4 cluster showed a high occurrence of pathways linked to chemotrophic carbon fixation and methane metabolism, nucleotide metabolism, valine, leucine, and isoleucine degradation, metabolism of cofactors and vitamins, translation, and folding, sorting, and degradation. The highest variations corresponded to the pathways of signal transduction and translation.

**Figure 6:**
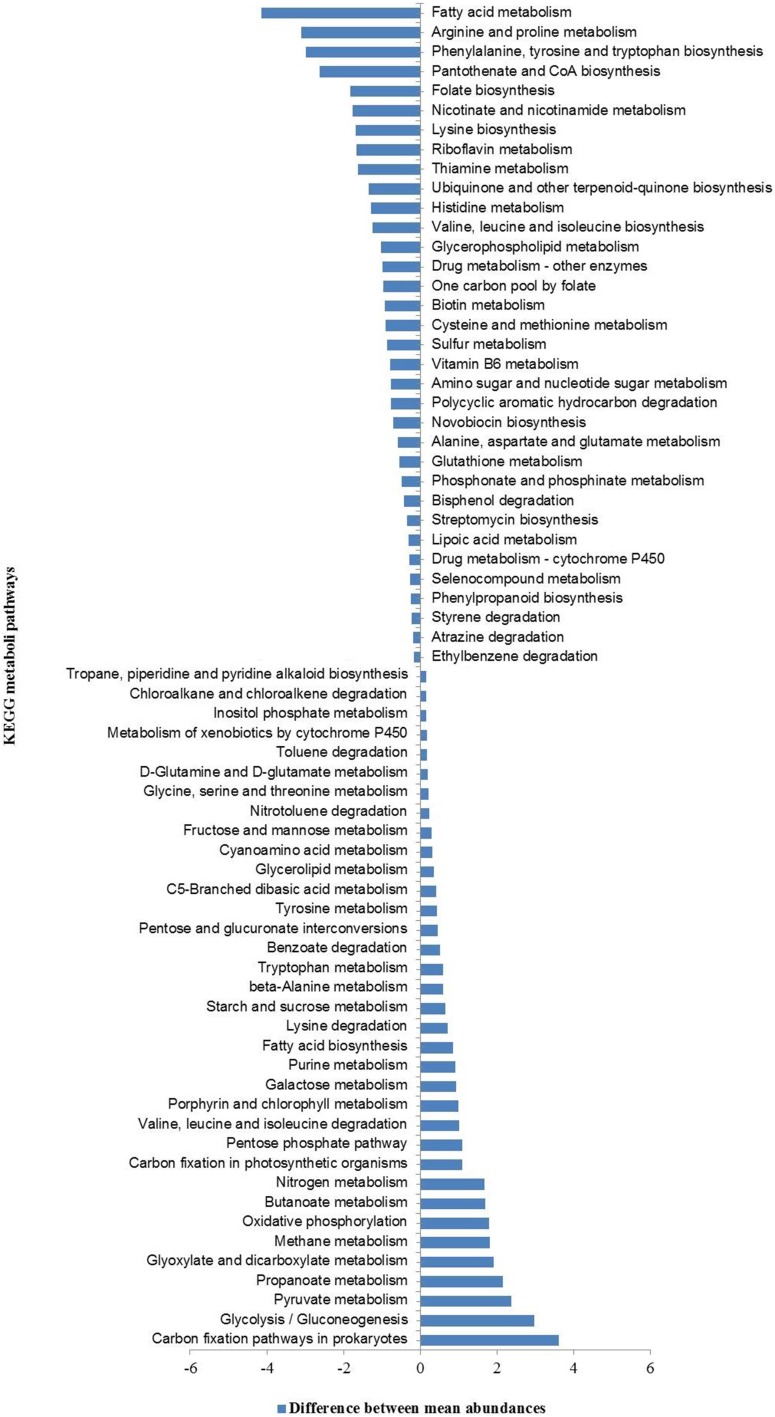
**KEGG pathways over- and under-represented in hot spring metagenomes relative to all sequenced bacterial and archaeal genomes (NCBI-RefSeq database)**.

Further, a comparison between the relative abundances of various functional pathways (KEGG classification) in our metagenomes and those in all sequenced bacterial and archaeal genomes (based on NCBI-RefSeq database) identified a set of 69 (sixty nine) functional pathways that were over- and/or under-represented. These included mostly pathways linked to the metabolism of carbohydrate, energy, amino acids, and xenobiotic biodegradation and metabolism (Figure 6).

### Linkage between microbial community function and environmental physicochemical parameters

The hierarchical clustering analyses showed that HT-1 and TP-2 metagenomes were functionally highly similar among the four metagenomes despite the wide geographical separation between the corresponding hot springs, and community composition and diversity. Previous studies have shown that functional compositions of the microbial communities are impacted by the prevailing physicochemical environmental conditions (Dinsdale et al., 2008; Raes et al., 2011). Canonical Correspondence Analysis (CCA) indicated direct associations between relative abundances of metabolic pathways and environmental physicochemical parameters (Supplementary Figure 8). The metabolic pathways of glutathione metabolism, and various xenobiotics degradation were found to be positively associated with the aqueous concentration of conductivity, fluoride, chloride and various metal ions (iron, arsenic, lead, and cadmium) whereas, the pathways of methane metabolism, one carbon pool by folate, glycolysis/gluconeogenesis, purine metabolism, terpenoid backbone biosynthesis, and nitrotoluene degradation were correlated with temperature. While relative abundance of genes related to nitrogen metabolism was positively associated with ammonia-nitrogen concentration, sulfur metabolism was positively correlated with sulfate concentration. Similarly, while the metabolic pathways of photosynthesis, porphyrin and chlorophyll metabolism, selenocompound metabolism and oxidative phosphorylation were positively associated with concentrations of magnesium, calcium, silicate-silicon, and total hardness of the springs, the pathways of citrate/TCA cycle, pyruvate metabolism, propanoate and butanoate metabolism, nicotinate and nicotinamide metabolism, carbon fixation pathways in prokaryotes and valine, leucine and isoleucine degradation were positively associated with the aqueous concentrations of zinc, nitrate-nitrogen and bicarbonate.

Overall, this analysis indicated that the environmental physicochemical parameters, such as aqueous concentration of metal ions (sodium, calcium, magnesium, iron, etc.), fluoride, chloride, sulfate, bicarbonate, total hardness, ammonia-nitrogen and nitrate-nitrogen, and conductivity, beside controlling the microbial composition and diversity, also plays important role in the dispersal of biological functions and adaptive responses of the communities in these hot spring habitats.

### Functional features of candidate phylum acetothermia

A unique feature of the spring TB-3 was the high abundance of members of the candidate phylum Acetothermia; roughly 24% of total assigned sequences in the TB-3 metagenome were affiliated to this group. The average G + C content of 55 ± 3% was approximately same as previously reported for Acetothermia bacterium (Rinke et al., 2013). The candidate phylum Acetothermia originally proposed as “candidate division OP1” (Hugenholtz et al., 1998), is a recently discovered lineage of bacteria. This candidate phylum is predicted to be one of the earliest evolved thermophilic chemolithoautotrophic bacterial lineages, encoding a nearly complete folate dependent acetyl-CoA pathway for carbon fixation and acetogenesis (Takami et al., 2012). In order to investigate the relatedness of the Acetothermia gene sequences retrieved in this study (form the spring TB-3) with the previously reported genome of “*Candidatus* Acetothermum autotrophicum” (Takami et al., 2012) and “Acetothermia bacterium SCGC AAA255-C06” (Rinke et al., 2013), we performed a detailed BLAST-based similarity search. Analysis of the BLAST result revealed that the majority of the hits to genes in our Acetothermia sequences primarily originated from the “*Ca*. A. autotrophicum” genome (88.7%), and only small percentage of the hits to genes originated from the “Acetothermia bacterium SCGC AAA255-C06” genome (7.8%) (Supplementary Figure 9A). Around 3.5% of the Acetothermia sequences in this study showed hits to both the published genomes; however, upon analyzing the identity ranges of the hits obtained with “*Ca*. A. autotrophicum” genome, it was revealed that only around 30% of these hits were having identities greater than 90% (Supplementary Figure 9B). Thus, it indicated that the Acetothermia-related gene sequences retrieved from the spring TB-3 probably originated form some hitherto uncharacterized members of the candidate phylum Acetothermia and were closely related to bacterium “*Candidatus* Acetothermus autotrophicum.”

**Figure 7:**
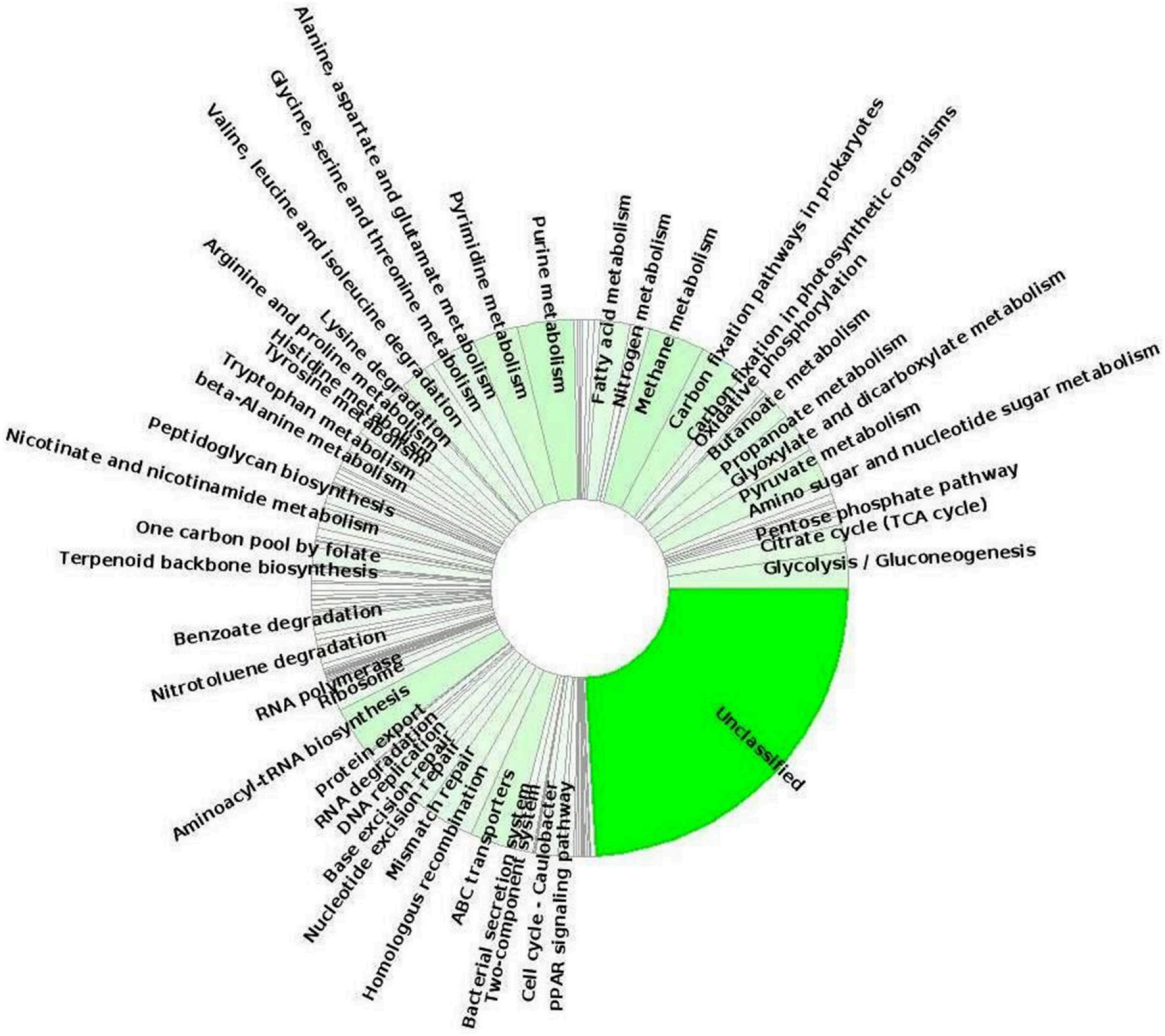
**Relative distribution of predicted protein-coding genes sequences that matched various KEGG pathways and were phylogenetically affiliated to Candidate phylum Acetothermia within the TB-3 metagenome sequence obtained using MEGAN5 software**.

Moreover, to gain more insight of the possible metabolic/functional capabilities of this candidate phylum of *Bacteria*, we looked at gene sequences encoding functions associated with different SEED subsystems and KEGG pathways. In this study, we detected multiple putative genes involved in carbon-fixation pathways, such as acetyl-CoA pathway (marker gene: acetyl-CoA synthase/CO dehydrogenase) and partial citric acid cycle/TCA cycle which were previously reported for Acetothermia bacterium (Takami et al., 2012). KEGG function annotation identified the methylmalonyl-CoA mutase subunit (EC:5.4.99.2; *mcmA1* and *mcmA2*) genes which were predicted to be involved in the archaeal-type autotrophic 3-hydroxypropionate/4-hydroxybutyrate cycle; however, a deeper analysis of the complete genomes of Acetothermia bacteria is required to confirm this observation. Analysis of the annotated metagenomic sequences detected several known gene involved in the pathways of energy metabolism, such as oxidative phosphorylation, glycolysis/gluconeogenesis, pyruvate metabolism, propionate, and butanoate metabolism, one carbon metabolism (serine-glyoxylate cycle) and fatty acid metabolism (Figure 7). These bacteria likely possess the pathways for degradation and transport of branched-chain amino acids: valine, leucine, and isoleucine, which were predicted to provide acetyl-CoA for the partial TCA cycle feeding various anabolic pathways under nutrient limiting conditions. Moreover, these bacteria contain both aerobic and anaerobic gene pair for pyruvate metabolism: pyruvate dehydrogenase (aerobic enzyme; EC 1.2.4.1) and pyruvate:ferredoxin oxidoreductase (anaerobic enzyme; EC 1.2.7.1) in addition to the genes for multiple electron transport complexes, and indicated that mixotrophy may be possible under certain growth conditions. Further, genes involved in the pathways for biopolymer degradation (endoglucanase enzyme, EC:3.2.1.4; beta-glucosidase, EC:3.2.1.21), synthesis and degradation of secondary metabolites (esterase/lipase, EC:3.1.1.-), and xenobiotics degradation like benzoate degradation, nitrotoulene degradation, ethylbenzene degradation, and drug metabolism-other enzymes (carboxylesterases, EC:3.1.1.1, 3.1.1.84) were also identified. The identification of a nearly full complement of genes related to the pathways of aminoacyl-tRNA biosynthesis indicated that the Acetothermia bacteria possibly biosynthesize all amino acids and are not dependent upon exogenous sources of these metabolites. Several genes associated with virulence (including resistance to antibiotics and heavy metals/toxic compound, Ton and Tol transport systems, and iron scavenging mechanisms), ABC-type membrane transport (transporters of sulfonate/nitrate/taurine, phosphate/phosphonate, zinc, iron, peptide/nickel, and cobalt), stress response, and DNA repair were also identified. The identification of the mviN genes predicted MviN flippase as the lipid-linked precursor for cell wall peptidoglycan biosynthesis in these bacteria. Thus, the members of the thermophilic candidate phylum Acetothermia are predicted to possess a versatile metabolism for survival in oligotrophic thermal environments, such as hot springs.

In conclusion, we report the metagenomic survey of four tropical hot springs located along the east coast in the state of Odisha, India to assess and understand the phylogenetic and functional diversity of microbial communities in these hot springs.

### Conflict of interest statement

The authors declare that the research was conducted in the absence of any commercial or financial relationships that could be construed as a potential conflict of interest.
