# Supplementary material for: Taxonomic and functional characteristics of microbial communities and their correlation with physicochemical properties of four geothermal springs in Odisha, India
Source: Front Microbiol. 2015 Oct 26;6:1166. doi: 10.3389/fmicb.2015.01166 (PMC4620158; doi:10.3389/fmicb.2015.01166)
Supplement: Supplementary file 1 [file DataSheet1.PDF]

Supplementary Material

Taxonomic and functional characteristics of microbial communities and their correlation with physicochemical properties of four geothermal springs in Odisha, India

Jhasketan Badhai[1], Tarini Shankar Ghosh[2] and Subrata K Das[1*]

[1]Department of Biotechnology, Institute of Life Sciences, Nalco Square, Bhubaneswar, India
[2]TATA Consultancy Services Limited, Bhubaneswar, India

[*]Corresponding author. Mailing address:
Institute of Life Sciences
Department of Biotechnology
Nalco Square, Bhubaneswar 751023 India
E-mail: subratkdas@hotmail.com / subrata@ils.res.in
Phone: (+91) 674 230 3342
Fax: (+91) 674 230 0728

Supplementary Table 1: Roche 454 GS-FLX pyrosequencing data.

**Sampling site**	**HT-1**	**TP-2**	**TB-3**	**AT-4**
Summary of QC-filtered 454 sequenced reads:				
Total no. of 454 reads	27,032	31,388	51,818	51,420
No. of high quality reads	26,423	30,644	50,543	49,872
Summary of CD-Hit454 duplicate removal:				
No. of unique reads	25,307	29,164	48,251	47,409
Total sequenced bases	11,589,561	13,289,605	22,001,653	21,803,188
Longest sequence	727	787	900	867
Shortest sequence	65	65	65	65
Average length	458	455	456	459
% GC distribution	10-85 %	10-85 %	15-90 %	10-85 %
Average % GC content	57±9%	57±11%	55±11%	55±9%
Taxonomic and Functional assignment of reads:				
NCBI-Taxonomy matches	15,167 (59.9%)	16,340 (56%)	27,706 (57.4%)	24,444 (51.6%)
SEED matches	7,249 (28.6%)	6,979 (23.9%)	13,726 (28.4%)	12,841 (27.1%)
KEGG matches	9,473 (37.4%)	9,560 (32.8%)	18,414 (38.2%)	17,213 (36.3%)

Supplementary Table 2: Number of sequences obtained for each phylum under the domains Bacteria and Archaea in each microbial community. Values in parentheses represent the relative abundance (%) of given taxa.

**Phylum**	**HT-1**	**TP-2**	**TB-3**	**AT-4**
Actinobacteria	103 (0.99)	194 (1.86)	144 (0.78)	169 (1.19)
Aquificae	233 (2.24)	0	51 (0.28)	47 (0.33)
Armatimonadetes	229 (2.20)	95 (0.91)	96 (0.52)	47 (0.33)
Bacteroidetes	264 (2.54)	494 (4.74)	477 (2.58)	131 (0.92)
Chlorobi	26 (0.25)	21 (0.20)	32 (0.17)	18 (0.13)
Ignavibacteriae	104 (1.00)	766 (7.35)	108 (0.58)	33 (0.23)
Verrucomicrobia	186 (1.79)	338 (3.24)	662 (3.58)	165 (1.16)
Chloroflexi	2831 (27.24)	1273 (12.21)	2188 (11.83)	6450 (45.50)
Cyanobacteria	90 (0.87)	1568 (15.05)	283 (1.53)	145 (1.02)
Deferribacteres	22 (0.21)	0	0	20 (0.14)
Deinococcus-Thermus	708 (6.81)	323 (3.10)	620 (3.35)	110 (0.78)
Dictyoglomi	10 (0.10)	0	0	41 (0.29)
uncultured bacterium	67 (0.64)	175 (1.68)	100 (0.54)	109 (0.77)
Acidobacteria	767 (7.38)	456 (4.38)	824 (4.45)	145 (1.02)
Marinimicrobia	0	20 (0.19)	0	29 (0.20)
Firmicutes	309 (2.97)	308 (2.96)	595 (3.22)	882 (6.22)
Gemmatimonadetes	95 (0.91)	19 (0.18)	55 (0.30)	0
Nitrospirae	235 (2.26)	48 (0.46)	644 (3.48)	1624 (11.46)
Planctomycetes	106 (1.02)	245 (2.35)	183 (0.99)	66 (0.47)
Proteobacteria	2494 (24.00)	3540 (33.97)	3270 (17.67)	1413 (9.97)
Spirochaetes	339 (3.26)	88 (0.84)	35 (0.19)	107 (0.75)
Synergistetes	0	15 (0.14)	19 (0.10)	23 (0.16)
Thermodesulfobacteria	0	0	21 (0.11)	35 (0.25)
Thermotogae	461 (4.44)	0	43(0.23)	829 (5.85)
Acetothermia	385 (3.70)	13 (0.12)	6849 (37.02)	264 (1.86)
Aminicenantes	34 (0.33)	42 (0.40)	164 (0.89)	39 (0.28)
Atribacteria	12 (0.12)	17 (0.16)	90 (0.49)	153 (1.08)
Calescamantes	0	0	0	28 (0.20)
Candidatus Saccharibacteria	0	13 (0.12)	0	0
Cloacimonetes	0	23 (0.22)	52 (0.28)	112 (0.79)
Fervidibacteria	43 (0.41)	11 (0.11)	55 (0.30)	32 (0.23)
Hydrogenedentes	38 (0.37)	12 (0.12)	33 (0.18)	56 (0.40)
Latescibacteria	0 (0.00)	18 (0.17)	0	18 (0.13)
Poribacteria	18 (0.17)	21 (0.20)	31 (0.17)	17 (0.12)
Crenarchaeota	21 (0.20)	10 (0.10)	255 (1.38)	185 (1.30)
Euryarchaeota	152 (1.46)	256 (2.46)	393 (2.12)	574 (4.05)
Korarchaeota	0	0	20 (0.11)	24 (0.17)
Thaumarchaeota	11 (0.11)	0	111 (0.60)	37 (0.26)

Supplementary Table 3: The Partial Least Square regression analysis between the relative abundances of twenty-two major genera as response variables and the twenty-two environmental parameters as the predictor variables. The first sheet contains the correlation coefficients along with their statistical significance (p-values) and the power of the correlation analysis (the power is indicative of how good the correlations are expected to be true).

Supplementary Table 4: SEED functional profiles (at the level of categories, subsystems and functional genes) generated from the MEGAN5 classification.

Supplementary Table 5: KEGG functional profiles (at the level of individual categories, pathways, and KEGG Orthology identifiers) generated from the MEGAN5 classification.

Supplementary Table 6: Distribution of identified genes involved in carbon fixation pathways from the four metagenomes.

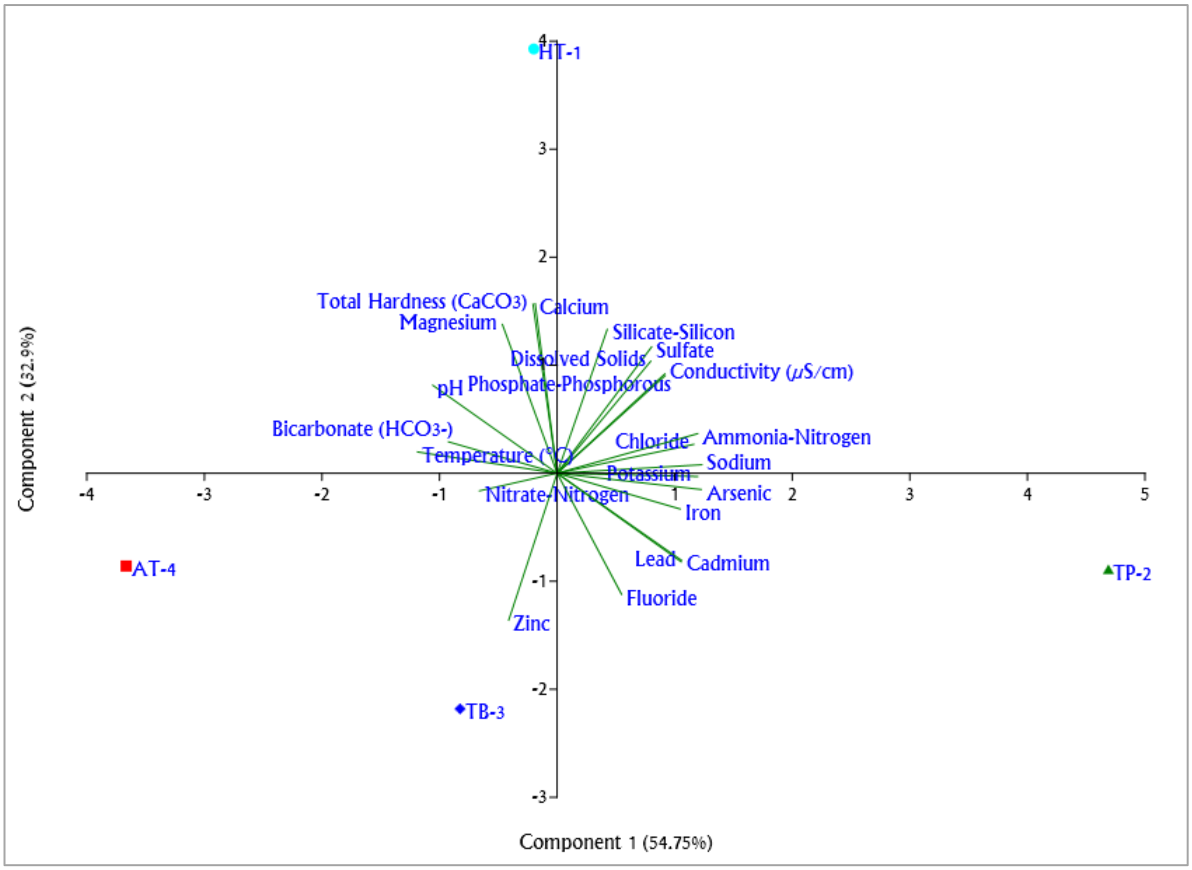

Supplementary Figure 1: Bioplot generated for the Principal Component Analysis (PCA) of twenty two physicochemical variables. Hot springs are shown as colored symbols and physicochemical variables are represented by green lines.

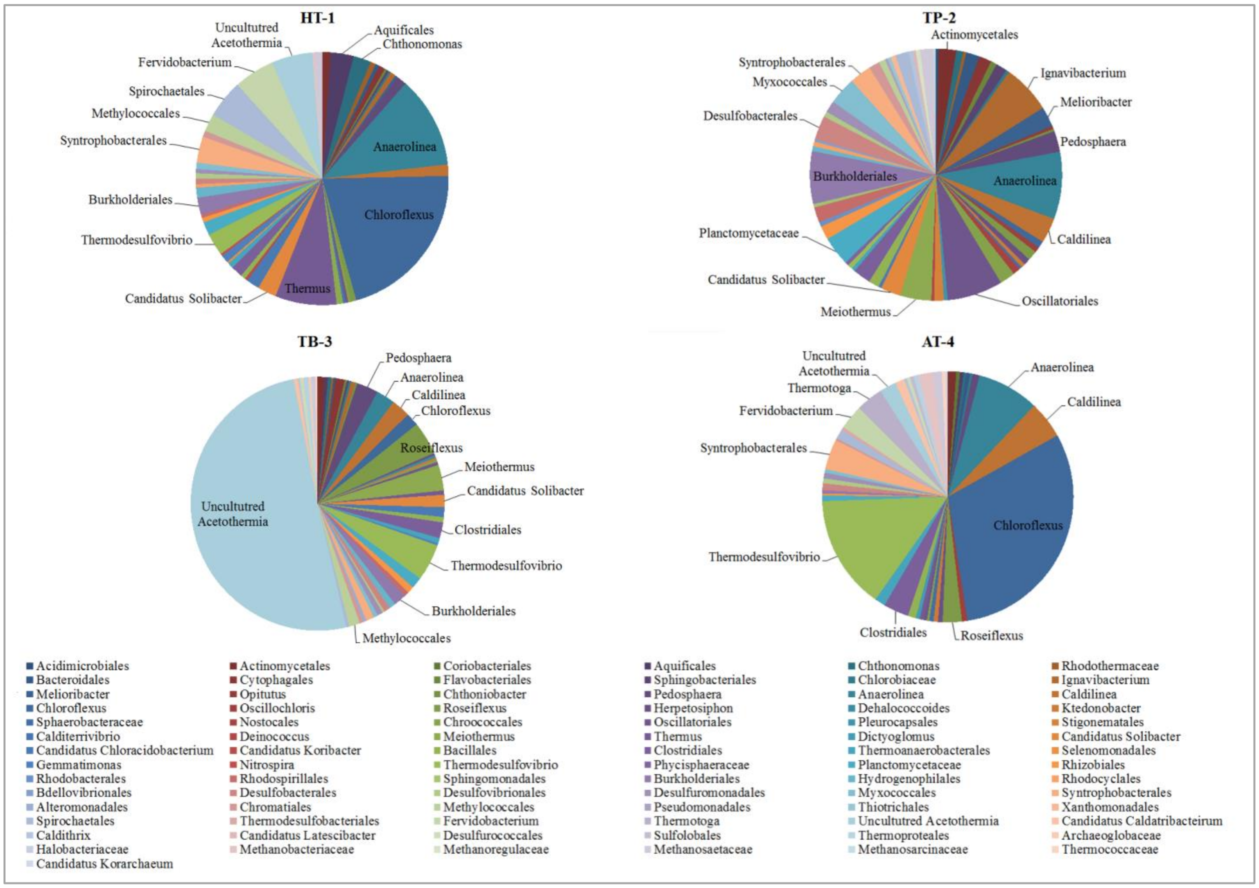

Supplementary Figure 2: Relative distribution of taxa (below phylum level) belonging to *Bacteria* and *Archaea*. The proportion of phyla is based on MEGAN5 taxonomic classification of the protein-coding sequences in the metagenomes: HT-1 (Athamallik), TP-2 (Taptapani), TB-3 (Tarabalo), and AT-4 (Atri).

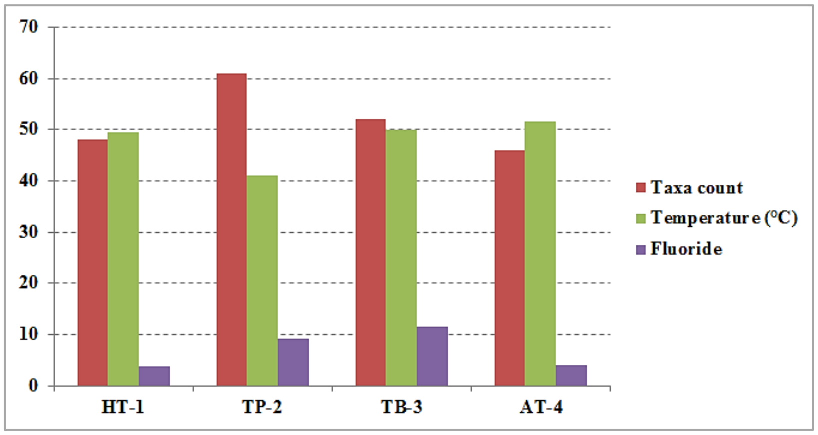

Supplementary Figure 3: Comparison of taxonomic richness with temperature (average) and aqueous concentration fluoride ions observed in the four hot springs: Athamallik (HT-1), Taptapani (TP-2), Tarabalo (TB-3) and Atri (AT-4).

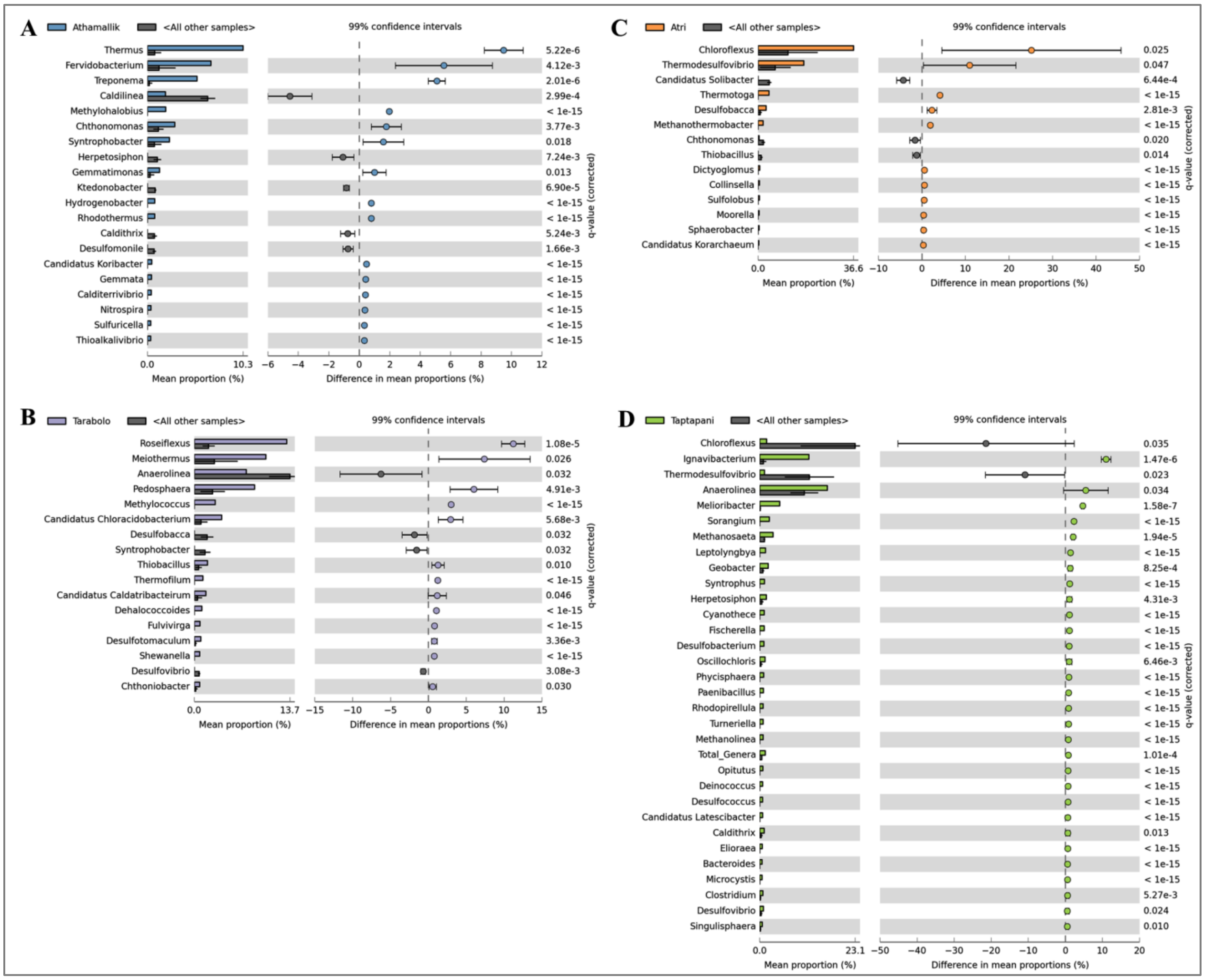

Supplementary Figure 4: Extended error bar plot showing genera significantly over-/under-represented in individual sample compared to the other three samples (cumulatively) are shown in (A) Athamallik *vs* all others, (B) Atri *vs* all others, (C) Tarabalo *vs* all others, and (D) Taptapani *vs* all others, respectively. The difference in proportions and the corrected p-value of significance are also indicated.

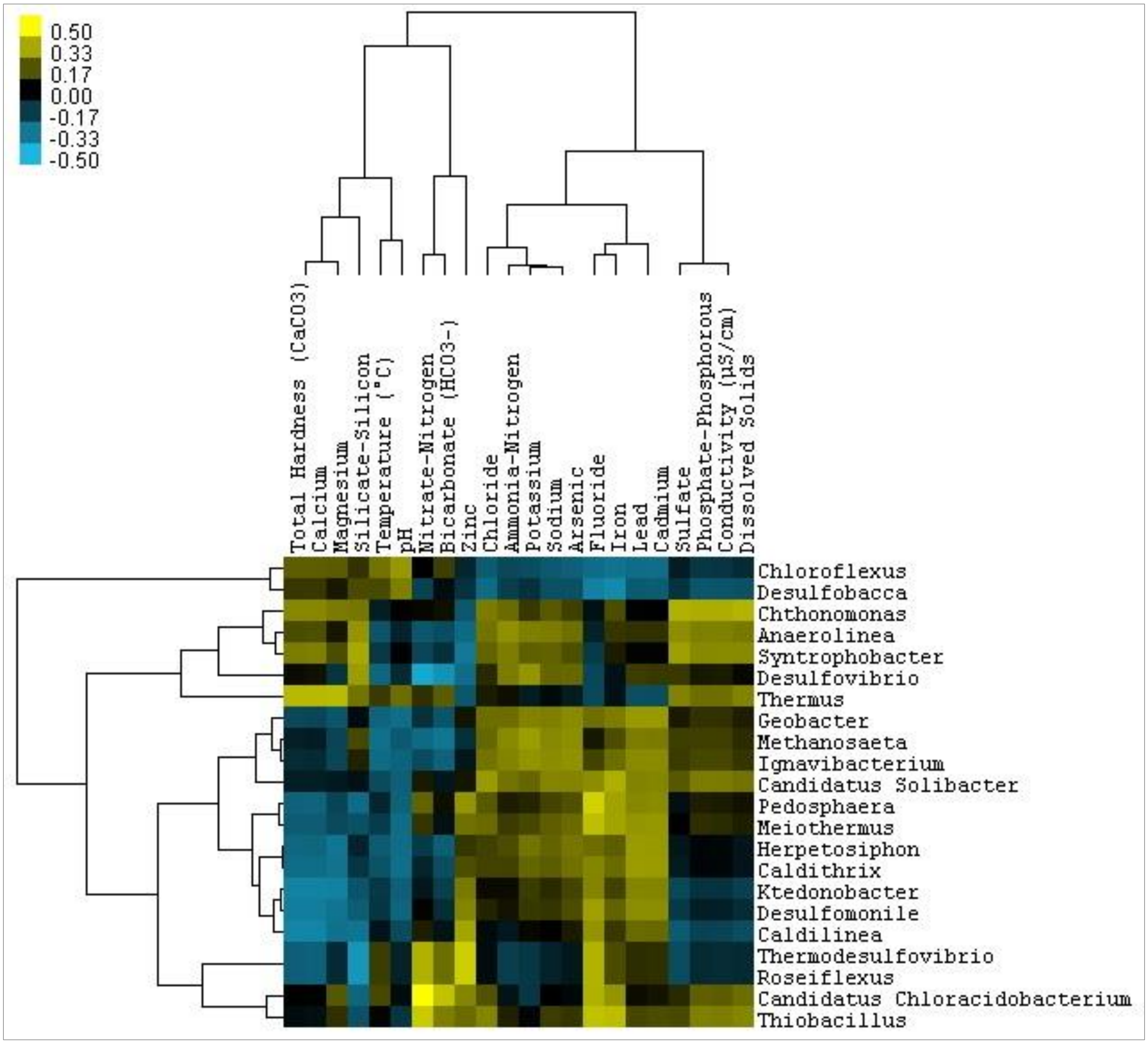

Supplementary Figure 5: Partial Least Square (PLS) regression analysis between relative abundances of major genera represented across at least three hot springs and the twenty two physicochemical parameters. The heatmap is based on the regression weights corresponding to the strength of influence an environmental variable has on the relative abundance of a given. Top dendrogram shows the clustering of the physicochemical parameters and side dendrogram shows the clustering of different genera.

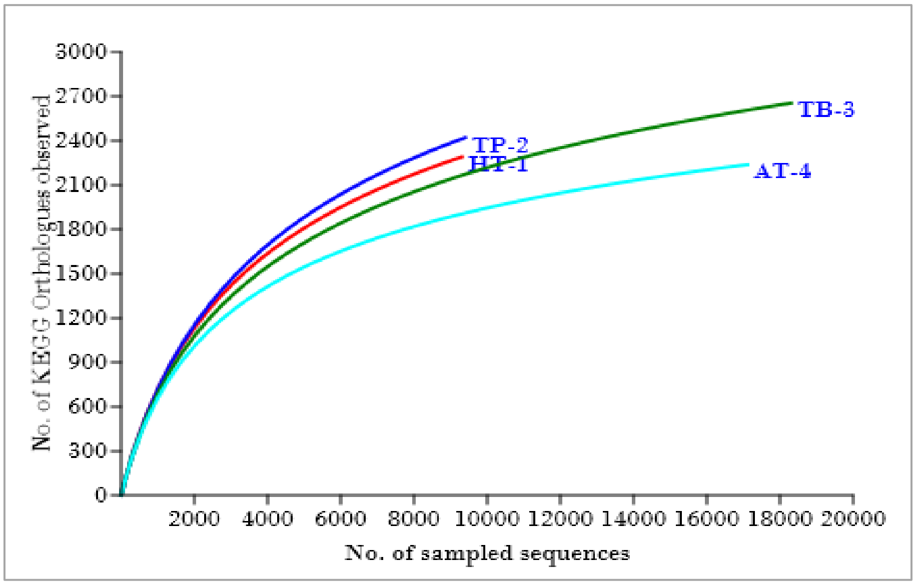

Supplementary Figure 6: Comparison of KEGG functional richness across metagenome samples based on rarefaction curves of unique KEGG Orthologous identifiers (KO-id) detected in each metagenome sample.

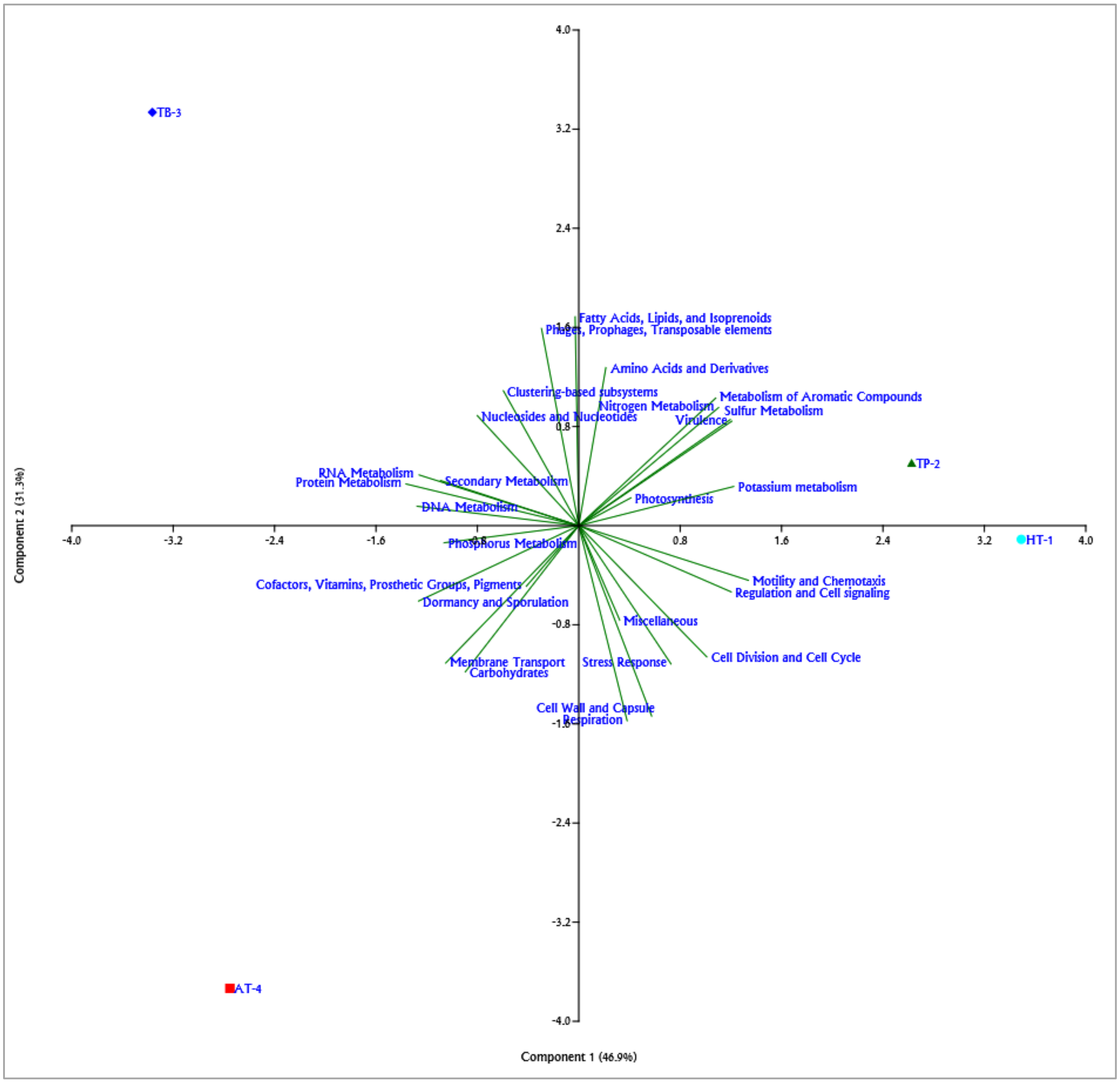

Supplementary Figure 7: Bioplot generated for the PCA of relative SEED categories represented across at least three hot spring samples. The green lines represent the SEED categories and the hot spring sites are shown as colored symbols.

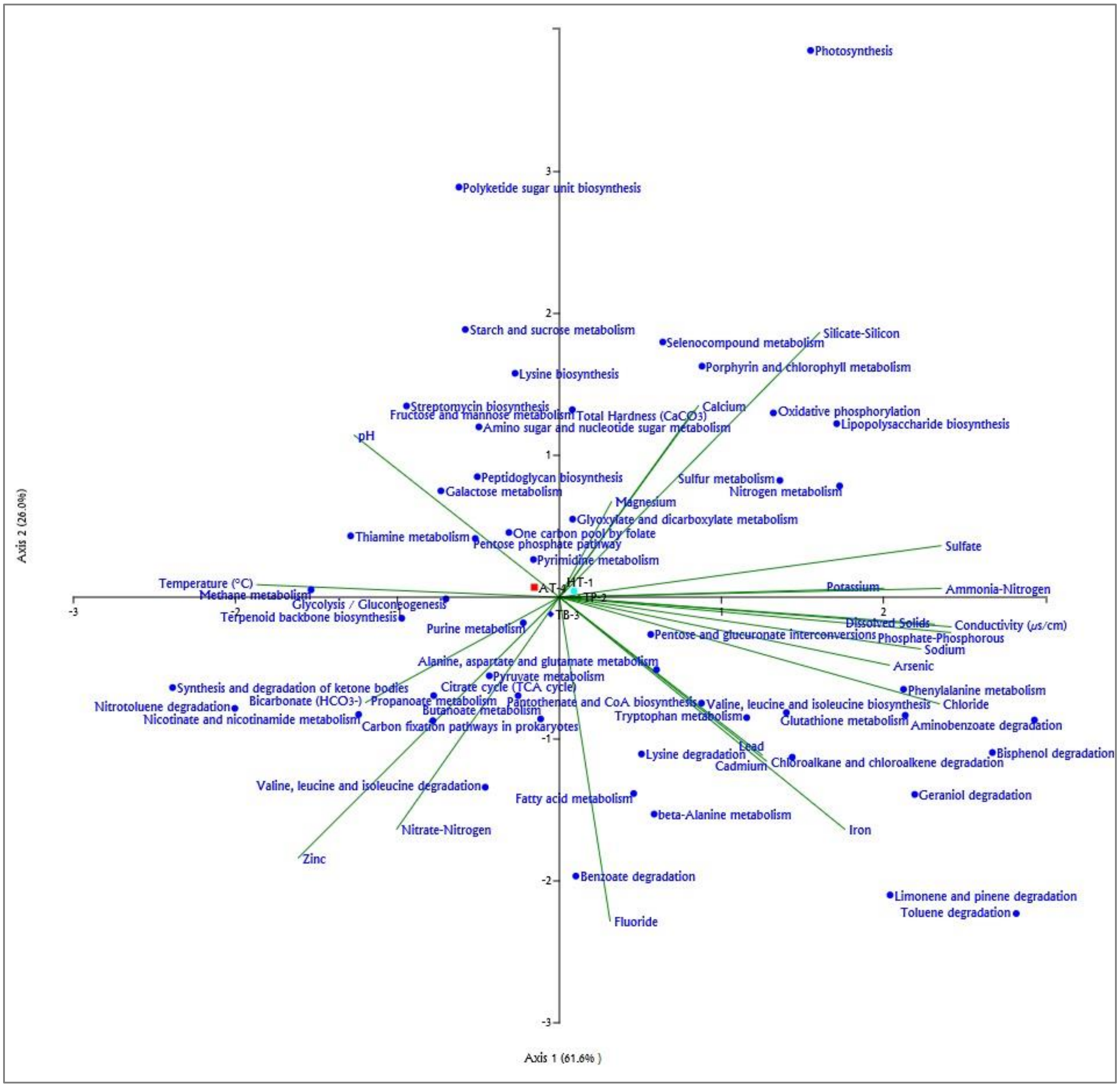

Supplementary Figure 8: Triplot generated for the Canonical Correspondence Analysis (CCA) of relative abundance of the top 50 most variable KEGG metabolic pathways (cut-off standard deviation of 10% within the pathway) and twenty two physicochemical parameters of the hot springs. The physicochemical parameters are represented by green lines; the KEGG pathways are shown as filled dots; hot springs: Athamallik (HT-1), Taptapani (TP-2), Tarabalo (TB-3) and Atri (AT-4) are shown as colored symbols.

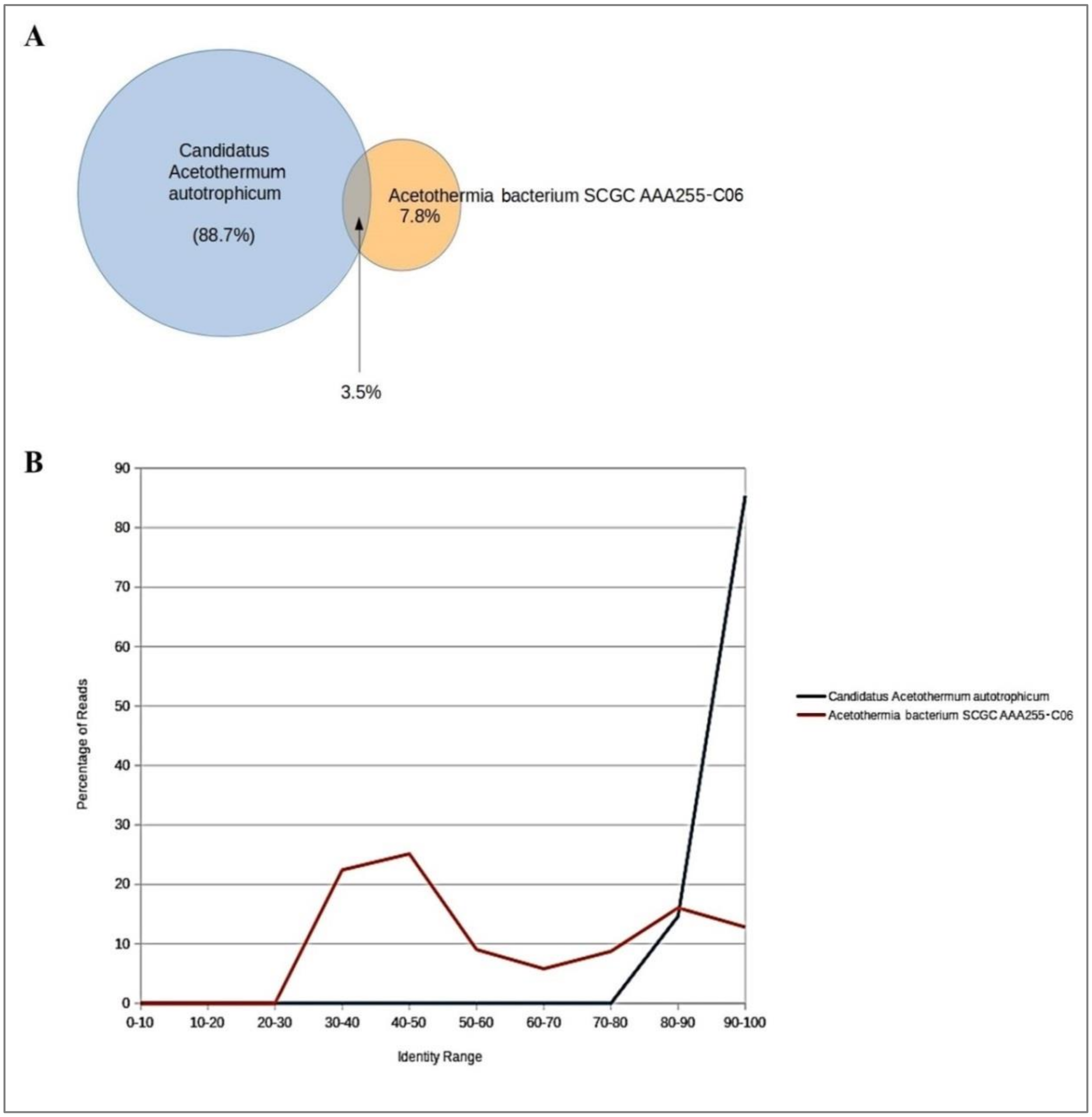

Supplementary Figure 9: Genomic relatedness between the candidate phylum Acetothermia affiliated read sequences present in the metagenome sample obtained from the spring TB-3 and the previously reported genomes of the bacteria "*Ca*. Acetothermum autotrophicum" and "Acetothermia bacterium SCGC AAA255-C06". **(A)** Venn-diagram showing percentage of hits to gene in Acetothermia sequences (obtained in this study) having matches in the other two genomes. **(B)** Distribution of percentage identities of the gene hits obtained from BLAST similarity search against the two published genomes of Acetothermia bacteria.
